# Mash-based analyses of *Escherichia coli* genomes reveal 14 distinct phylogroups

**DOI:** 10.1038/s42003-020-01626-5

**Published:** 2021-01-26

**Authors:** Kaleb Abram, Zulema Udaondo, Carissa Bleker, Visanu Wanchai, Trudy M. Wassenaar, Michael S. Robeson, David W. Ussery

**Affiliations:** 1 Department of Biomedical Informatics, University of Arkansas for Medical Sciences, Little Rock, Arkansas 72205 USA; 2 The Bredesen Center for Interdisciplinary Research and Graduate Education, University of Tennessee, Knoxville, Tennessee 37996 USA; 3 Department of Electrical Engineering and Computer Science, University of Tennessee, Knoxville, Tennessee 37996 USA; 4 Molecular Microbiology and Genomics Consultants, 55576 Zotzenheim, Germany

**Keywords:** Classification and taxonomy, Genome informatics

## Abstract

In this study, more than one hundred thousand *Escherichia coli* and *Shigella* genomes were examined and classified. This is, to our knowledge, the largest *E. coli* genome dataset analyzed to date. A Mash-based analysis of a cleaned set of 10,667 *E. coli* genomes from GenBank revealed 14 distinct phylogroups. A representative genome or medoid identified for each phylogroup was used as a proxy to classify 95,525 unassembled genomes from the Sequence Read Archive (SRA). We find that most of the sequenced *E. coli* genomes belong to four phylogroups (A, C, B1 and E2(O157)). Authenticity of the 14 phylogroups is supported by several different lines of evidence: phylogroup-specific core genes, a phylogenetic tree constructed with 2613 single copy core genes, and differences in the rates of gene gain/loss/duplication. The methodology used in this work is able to reproduce known phylogroups, as well as to identify previously uncharacterized phylogroups in *E. coli* species.

## Abstract (web summary)

Kaleb Abram and Zulema Udaondo et al. analyze over 100,000 publicly available *E. coli* and *Shigella* genome sequences and perform a Mash-based analysis to identify 14 unique phylogroups. Their results reveal that most of the sequenced *E. coli* genomes belong to four distinct phylogroups.

## Introduction

*Escherichia coli* is a common inhabitant of the gastrointestinal tract of warm-blooded organisms and can also be found in soil and freshwater environments^1^. The species comprised both commensal and pathogenic strains, which can cause disease in a wide variety of hosts^2^. For historical and medical reasons “*Shigella*” strains have kept a separate genus name, even though it has been well established for many years, based on a variety of methods, that *Shigella* are effectively a subspecies of *E. coli*^3^. In humans, pathogenic *E. coli* strains are a leading cause of diarrhea-associated hospitalizations^4^. Some of the reasons why *E. coli* is intensely studied are as follows: rapid growth rate in the presence of oxygen, easy adaptation to environmental changes, and the relative ease with which it can be genetically manipulated^5^. Genomic diversity of the species, to which the genus *Shigella* has been proposed to be included^6,7^, is reflected by the existence of several phylogenetic groups (phylogroups) that have been identified using a variety of different methods^8–10^.

Historically, four phylogroups have been recognized as detectable by triplex PCR: A, B1, B2, and D^8,10^, and three more were added later^11^: phylogroups C (closest relative to B1), F (as a sister group of phylogroup B2), and E to which many D members were reassigned. Some studies have further subdivided these phylogroups with subdivisions of F and D, and separate phylogroups for *Shigella* species^12^. Recently, Clermont et al.^13^ characterized phylotype G using multiplex PCR as an intermediate phylogroup between B2 and F. These phylogroups are thought to be monophyletic^10,12^ and partially coincide with different ecological niches and lifestyles. Moreover, phylogroups differ in metabolic characteristics, the presence of virulence genes, and also in antibiotic resistance profiles^10,14–16^.

As bacterial genome sequencing is becoming easier, faster, and less expensive, several methods have been developed for whole-genome comparison. Thus, the phylogroups originally characterized by PCR and multilocus sequencing typing (MLST) and multilocus enzyme electrophoresis (MLEE) appear to be distinct phylogenetic clades, reproduced consistently from a variety of different whole-genome-based methods^12,17–19^.

Here we describe a comprehensive analysis of over 100,000 publicly available genome sequences, consisting of 12,602 assembled genomic sequences from GenBank and over 125,000 unassembled genome sequences from the Sequence Read Archive (SRA). This study combines whole-genome sequences (WGS) and SRA unassembled genomes using high-performance computing resources, to conduct, to our knowledge, the largest analysis to date of the population structure of *E. coli*. We have assessed the genomic similarities and differences between phylogroups to characterize the genetic heterogeneity of these different phylogenetic lineages. We have also identified 14 “medoid”^20^ genomes that can be considered as the genetic “center” of each of the phylogroups in our dataset and can be used as a representative sequence for the associated phylogroup. Furthermore, this study has application to the fields of public health and medical science, as it provides detailed information about the existing diversity of the *E. coli* species enabling public health researchers to identify pathogenic strains that belong to the same genetic lineage appearing in outbreaks at different temporal and geographical locations.

## Results

### Dataset description

A set of 12,602 genome sequences, labeled either *Escherichia* or *Shigella*, were downloaded from GenBank and cleaned to obtain an informative and diverse set of 10,667 *E. coli* and *Shigella* genomes (Supplementary Data 1). In addition to the GenBank genomes, a total of 125,771 unassembled read sets labeled as either *E. coli* or *Shigella* were downloaded from the SRA database (see “Methods”).

### Mash analysis of *E. coli* genomic sequences reveals 14 phylogroups

**Fig. 1:**
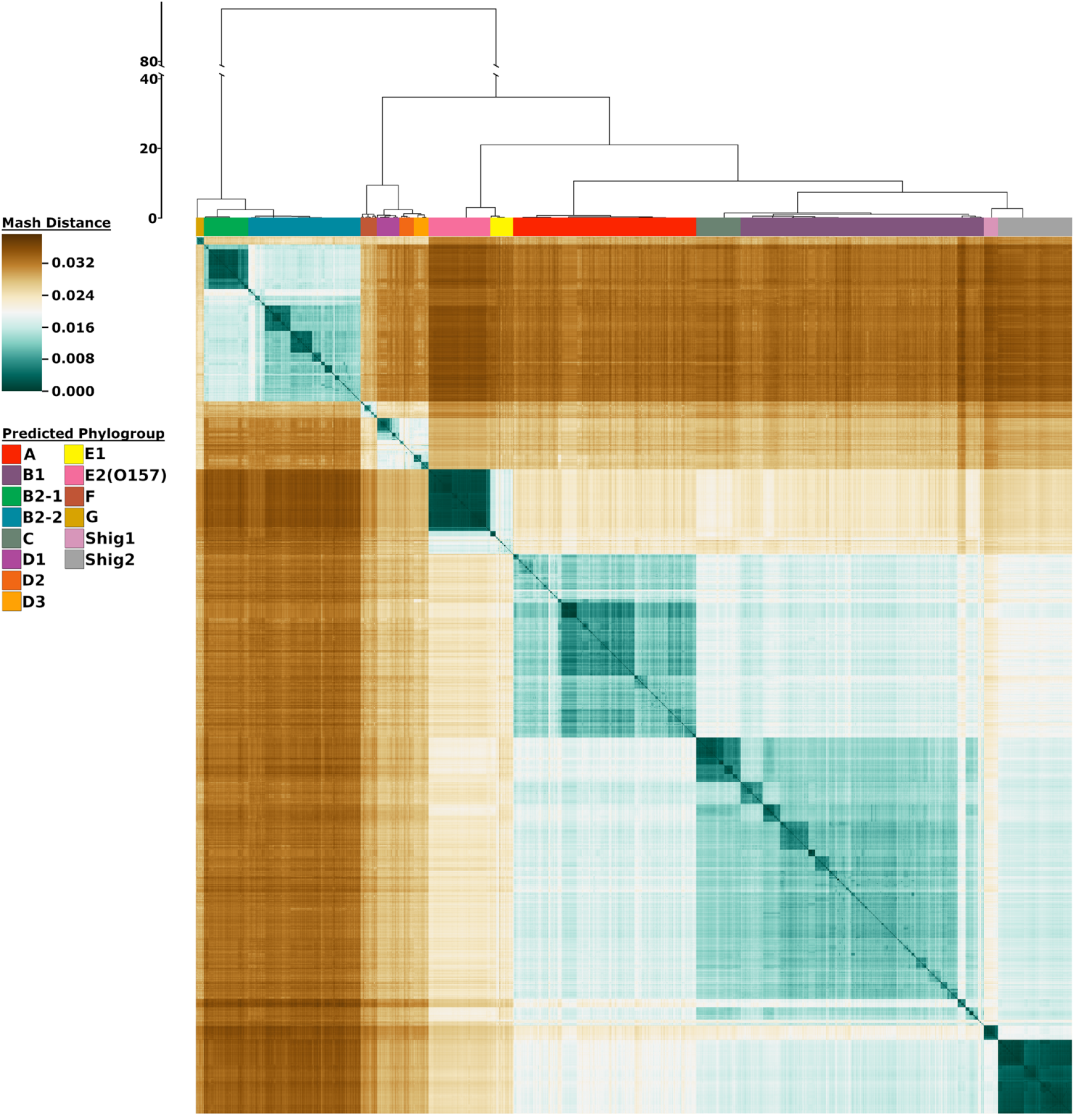
Heatmap representation of 10,667 genomes using Mash distances. The color bars at the top of the heatmap identify the phylogroups as predicted from the analysis. The scale to the left of the dendrogram corresponds to the resultant cluster height of the entire dataset obtained from hclust function in R. The colors in the heatmap are based on the pairwise Mash distances. Shades of teal represent similarity between genomes, with the darkest teal corresponding to identical genomes reporting a Mash distance of 0. Shades of brown represent low genetic similarity per Mash distance, with the darkest brown indicating a maximum distance of ~0.039. Genomes of relative median genetic similarity have the lightest color.

We utilized Mash^21^, a program that approximates similarity between two genomes in nucleotide content, and an in-house Python script to create a matrix of distances for all 10,667 genomes. This matrix was then clustered using hierarchical clustering to produce a heatmap, which illustrates the population structure of these genomes (Fig. 1). This methodology differentiated 14 different phylogroups, consisting of 12 *E. coli* groups: G, B2-1, B2-2, F, D1, D2, D3, E2(O157), E1, A, C, and B1, and 2 *Shigella* groups: Shig1 and Shig2 (ordered as shown in Fig. 1). These groups were determined by using a cutoff, allowing the most recently accepted phylogroup in the literature (Phylogroup C) to split off. All genomes within each of these individual phylogroups share a lower intragroup distance (meaning they display a higher genetic similarity) than they do to any other genome within the rest of the dataset. The phylogroups Shig1 and Shig2 exclusively contained *Shigella* species, but *Shigella* sp. genomes were also found in phylogroups A, B1, B2-2, D2, D3, E1, and F (Supplementary Fig. 1). In addition, the genetic relatedness between any phylogroup and the rest of the species is graphically shown. For example, phylogroups A, B1, and C are more genetically similar to each other than any one of these phylogroups are to B2-1 or B2-2, as illustrated by lower Mash distances between phylogroups A, B1, and C compared to B2-1 or B2-2. Figure 1 also illustrates the phylogroup substructure or intragroup genetic relatedness. E2(O157), Shig1, and Shig2 are the most homogeneous, which can be seen by the limited range of Mash distances within these phylogroups (Supplementary Fig. 2). This suggests that these phylogroups contain relatively large fractions of clonally related genomes. On the other hand, B1 and B2-2 are more heterogeneous as shown by numerous smaller dark teal squares that correspond to clusters of genomes that have a lower Mash distance between them compared to the rest of the genomes in that phylogroup. The relative abundance of phylogroup sequences can also be compared in Fig. 1. Phylogroup G has the smallest number of sequenced genomes and B1 has the largest number of sequenced genomes in the assembled dataset.

Microreact^22^, a web application developed to dynamically view genetic trees and associated metadata, was utilized to further explore the results of the Mash-based analysis, as this provides an easy medium for researchers to determine the closest genetic neighbors to any genome in this dataset. To leverage the search function of Microreact for our dataset, we mapped and displayed all available metadata found for our dataset from the database PATRIC^23^ (downloaded on 20 June 2019). Further, MLST and comparative analysis leveraging ClermonTyping methods^24,25^ were also performed and are shown along with Boolean comparison from the results of ClermonTyping to the results of our methodology. In addition, due to the inclusion of some clinically relevant outbreak strains, such as O157:H7, O104:H4, and O104:H21, basic retroactive genomic surveillance is possible by identifying strains of known outbreaks and noting their nearest neighbors. This data is available on Microreact at: https://microreact.org/project/10667ecoli/c38356ec.

### Currently sequenced *E. coli* and *Shigella* species can be represented by 14 medoid genomes

**Fig. 2:**
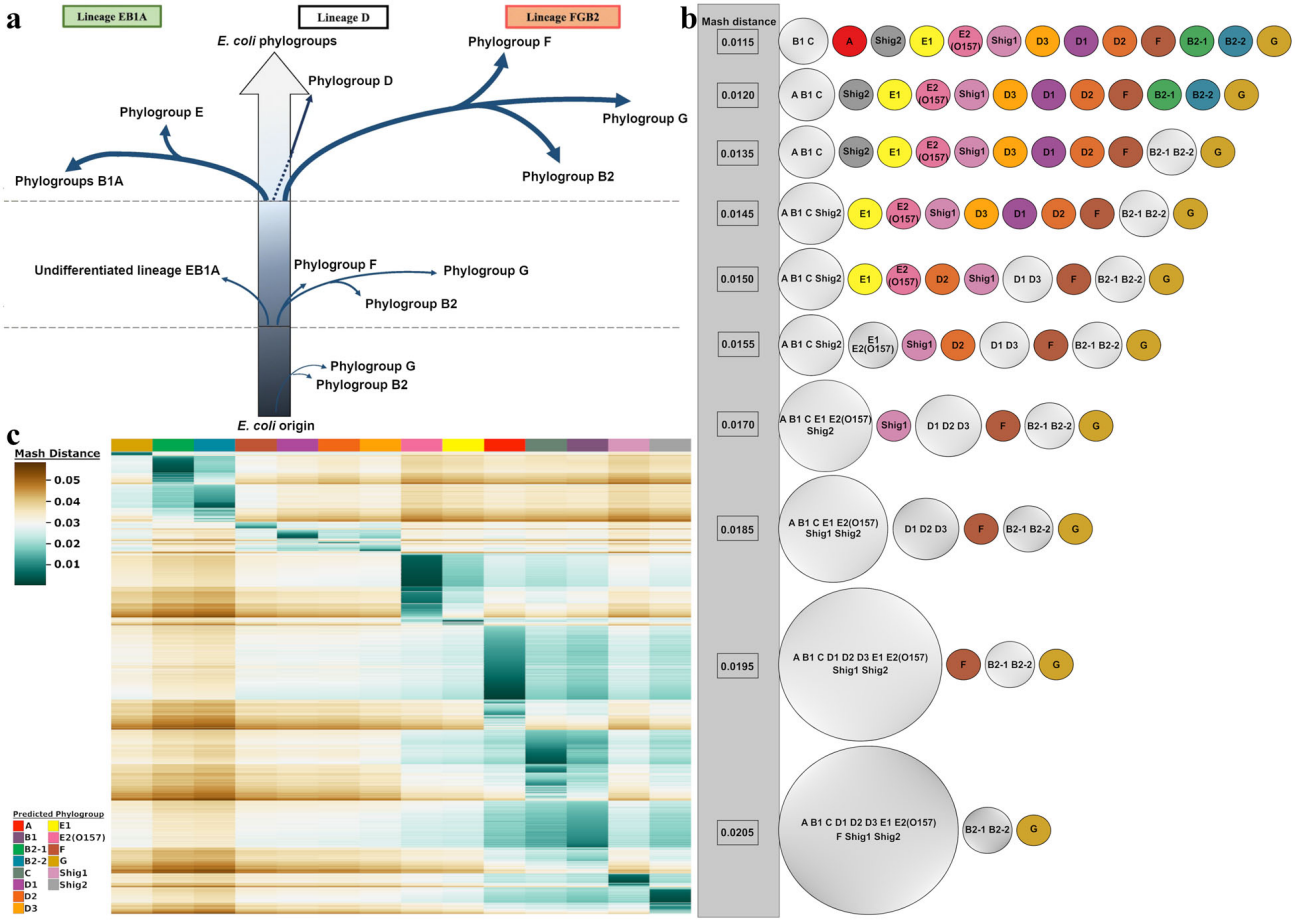
Summary of phylogroup differentiation and heatmap representation of sequence reads from the SRA database. **a** Evolutionary scenario in the diversification of *E. coli* adapted from Gonzalez-Alba et al.^19^, based on their methodology “SP-mPH,” a combination of “stratified phylogeny” and “molecular polymorphism hallmark.” Each branch reflects SNPs accrued by each phylogroup over time. Branch length is not proportional to the observed evolutionary distance. **b** Summary of the Cytoscape analysis. Phylogroups are colored based on the same color scheme in Fig. 1. Phylogroups with more than one member are gray colored. The Mash distance at which each division occurs at is indicated by numerical value in the gray bar that runs down the side of this panel. **c** Clustered heatmap of 91,260 unassembled sequence reads. The heatmap colors are based on the pairwise Mash distance between the SRA read sets and the 14 medoid genomes, one for each phylogroup, which are presented in the same order as in Fig. 1. To be included, SRA reads sets had to have three or more medoid comparisons producing a Mash distance equal to or less than 0.04. This removed 4265 SRA read sets from the dataset. The number of SRA reads mapped to each medoids is given below the heatmap. Additional heatmaps of the SRA data can be found in Supplementary Figs. 3–16.

To increase the utility of our analysis, we tested whether a minimal set of genomes could be defined to represent the diversity of the 10,667 genomes without suppressing any of the predicted phylogroups. As 14 phylogroups were detected, we tested whether one genome from each of the phylogroups would be enough to accurately classify any genome sequence claiming to be *E. coli* or *Shigella* using our methodology. Each of these 14 genomes represents the medoid^18^ or the “genomic center” of each phylogroup based on the 10,667 analyzed assembled genomes. Furthermore, to investigate whether our clustering results were due to the data itself and not due to bias in hierarchical clustering methods, we utilized Cytoscape (version 3.7.1), an open source software platform for visualizing complex networks^26^. In this analysis, a sliding cutoff ranging from 0.04 to 0.0095 with a step size of 0.005 was used to isolate genomes to the medoid that they had the lowest Mash distance to. Thus, the medoids were used as anchors to evaluate how the rest of the genomes distributed amongst the phylogroups according to the relative genetic distances of the genomes as calculated by raw Mash values. Our results show high correspondence with the recently proposed evolutionary scenario for the *E. coli* species^19^ (Fig. 2a). The Cytoscape analysis showed that the two B2 phylogroups are the most genetically distinct from the remainder of the species as they separate earliest from the other phylogroups. At the final Mash value cutoff of 0.0095, the C and B1 phylogroups become the last two groups to separate. This last split is indicative of the relatively large shared genomic content between these two phylogroups. The resultant Cytoscape graphs were collected into a video available on figshare via 10.6084/m9.figshare.13105235 and a collection of stills is hosted on figshare via 10.6084/m9.figshare.11473308. An overview of the Cytoscape results is shown in Fig. 2b. Between the initial Cytoscape frame and the final frame, the number of genomes represented decreased by 43%, whereas the edges (connections between genomes and medoids) decreased by 96%. As the cutoff decreases, some genomes are no longer represented in the Cytoscape analysis due to having no Mash distance equal to or less than the applied cutoff. As expected, the overall interconnectivity between the different phylogroups drops significantly with the cutoff, but intraconnectivity within the phylogroups does not. Upon visualization and inspection of the data via Cytoscape, we could verify that each of the medoids is representative of its entire phylogroup. We propose that the 14 medoids are suitable as proxies to decrease visual complexity without sacrificing accuracy. Information about the 14 found medoids is available in Supplementary Table 1. A representation of the division of the phylogroups via raw Mash distances is shown in the movie available on figshare (10.6084/m9.figshare.13105235) and can be found in Fig. 2b.

### Most sequenced *E. coli* genomes belong to four phylogroups

The vast majority of *E. coli* genomic sequences are available as unassembled genomes deposited in the SRA database. To assess whether our medoids could be utilized to classify the phylogroup of raw sequence reads, thus expanding the scope of our methodology to unassembled sequences, we added a total of 125,771 read sets labeled as either *E. coli* or *Shigella* from the SRA database. This dataset was first filtered by sequence quality (see “Methods”), which resulted in a set of 95,525 genomes. As a way to reduce computational load for classifying SRA reads, these were compared to each medoid of the 14 phylogroups in an asymmetric matrix. Of the remaining 95,525 read sets, two-thirds (67%) belong to 4 phylogroups: A (23%), C (15%), B1 (15%), and E2(O157) (14%) (Fig. 2c). This large disparity in phylogroup diversity in the SRA dataset most likely reflects the research interests of the scientific and medical communities. Strains belonging to phylogroups B1, C, and E2(O157) are often pathogenic and of interest to medical research and epidemiology, whereas phylogroup A includes strains frequently used in the laboratory (e.g., strain K-12) or genetically modified strains (such as strains BL21 and REL606). A different distribution was observed for the 10,667 assembled genomes, of which 70% belong to four phylogroups: B1 (28%), A (21%), B2-2 (13%), and Shig2 (8%). In the assembled genomes dataset, phylogroup C is represented by 5% and E2(O157) is by 7% of the genomes in that set. It is somewhat unexpected that the assembled genomes have a different distribution of genomes than the unassembled dataset; however, this could be due to the speed and low costs involved to produce unassembled genome reads, where their utility is sufficient for genomic surveillance of outbreaks. A breakdown of the results for the SRA analysis including the number of medoid hits below the cutoff is summarized in Supplementary Data 2. In addition, a collection of heatmaps with different membership cutoffs, ranging from 1 to 14 phylogroups, can be found in Supplementary Figs. 3–16.

### Members of Mash phylogroups possess different genomic features

As Mash values provide a measure of similarity via distance between pairs of genomes, the phylogroups of Fig. 1 are the consequence of differences/similarities in the genetic content of each genome with respect to the rest of the genomes included in the analysis. Differences in genome size and percentage of GC content between phylogenetic groups were observed for the 10,667 assembled genomes (Supplementary Fig. 17) and statistical tests were performed by analysis of variance (ANOVA) and Tukey’s multiple comparison test (see “Methods” and Supplementary Data 3), suggesting that genomes from phylogroups Shig1, Shig2, A, B1, and B2-1 are significantly smaller in size than those from phylogroups E2(O157) and C (*P* < 0.01). The smaller genome size of the strains from both *Shigella* phylogroups is indicative of a reductive evolution of the genomes of these strains as previously described^27^, which is mainly driven by their pathogenic nature. Other enteroinvasive *E. coli* strains such as serotypes O124, O152, O135, and O112ac were classified inside phylogroups A (which also contains engineered, lab, and commensal strains) and B1 (often containing commensal enteric strains), which are the most heterogeneous phylogroups due to the diverse nature of their strains in terms of their environmental niches. This heterogeneity is also reflected in the large ranges of genome size and GC content of these two phylogroups. However, reduced genome size is not associated with pathogenicity per se, as the large genomes of E2(O157) and C phylogroups illustrate. Larger genome sizes associated with virulence may result from the accumulation of virulence genes in prophages, pathogenicity islands, and plasmids^28^, such as the large genomes of E2(O157) and C phylogroup members illustrate. Significant genomic differences in GC content, with respect to other phylogroups were only found for the two *Shigella* phylogroups (*P* < 0.01), which also agrees with an ongoing purifying or negative selection occurring in these genomes^27^. These characteristics might reflect the different evolutionary strategies and opposite selection pressures as a consequence of adaptation to diverse niches in which the different phylogroups have evolved^29^.

### Level of preservation of homologous genes varies between phylogroups

**Fig. 3:**
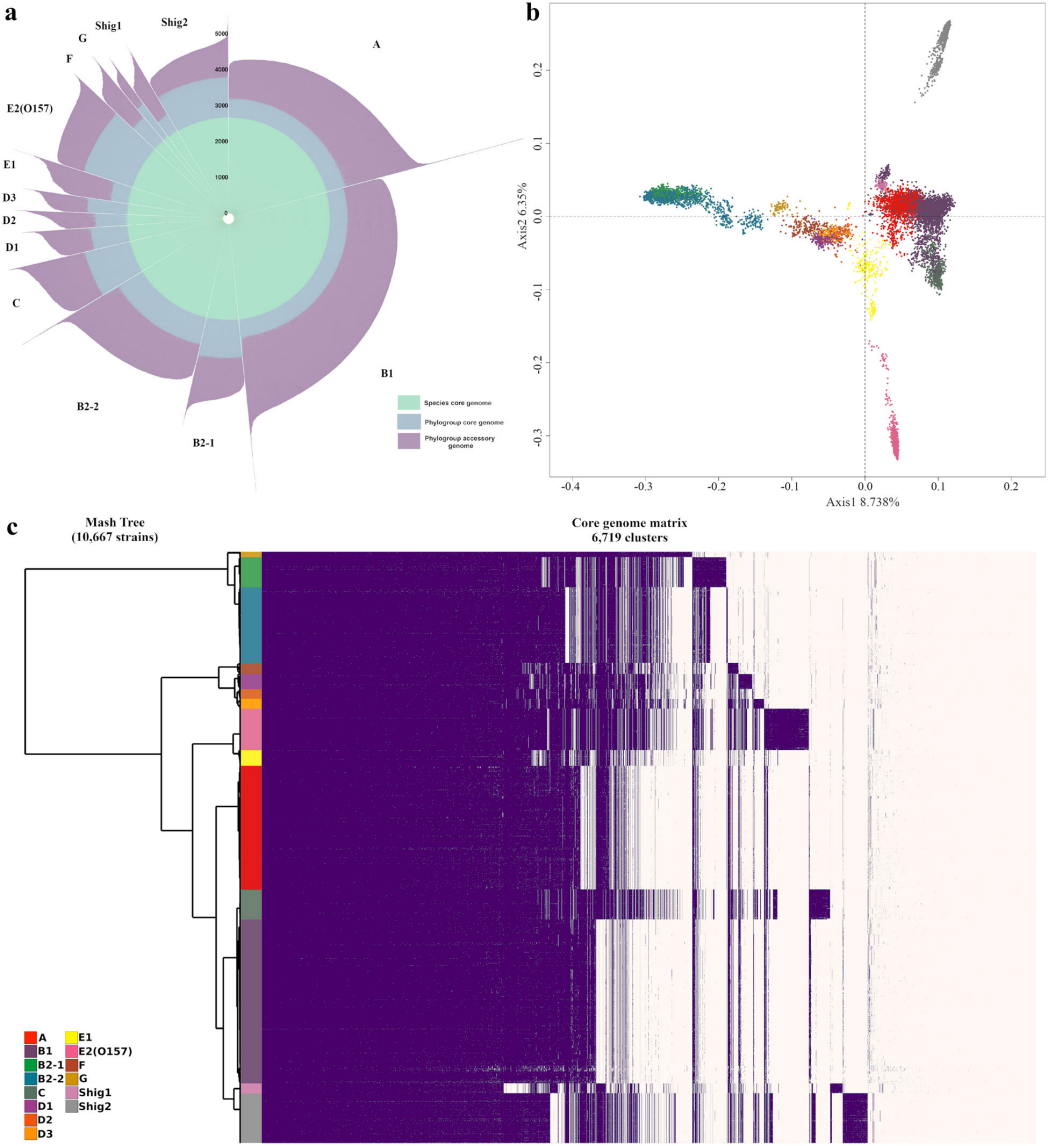
Pangenome representations of *E. coli* and *Shigella*. **a** Each bar length of the circular bar plot represents the total number of proteins of a single genome, grouped by phylogroup. The proteins belonging to the ^TOT^core_97_ genome are shown in green. Additional proteins shared in each ^PHY^core_97_ genome are shown in blue, whereas purple is reserved for accessory proteins. **b** Principal Coordinate Analysis plot of 135,983 protein families of 10,667 assembled genomes. Phylogroups are indicated by the same color scheme used in Figs. 1 and 2. **c** Core genome matrix of 6719 phylogroup core clusters and 10,667 assembled genomes. Clusters are sorted such that the core for the species is placed first, then the phylogroup core genes are placed, sorted by their overall abundance in the species for each phylogroup in the same order as Fig. 1; finally, the remaining clusters are placed by overall abundance. Phylogroup unique core genes are indicated by purple blocks which do not appear in other phylogroups.

To evaluate the existence of functional traits associated with each of the phylogroups, we conducted pangenome-approach based analyses using the proteomes of the 10,667 assembled genomes (translated from the respective genome sequences under a set of standardized conditions). In addition, separate pan and core genomes were calculated for the 14 individual phylogroups. This approach allowed us to highlight the unique proteomic cores of each phylogroup, which in turns helps to define their distinct biology. The total set of genes of the species (pangenome) is comprised of 135,983 clusters of homologous proteins (Table 1). By testing the cutoffs for core genome conservation from 90% to 99% of the genomes (Supplementary Fig. 18), we concluded that, although the traditional cutoff for core genome calculation of 95% of genomes would suffice, a cutoff of 97% can minimize erroneous false positive core genes thus providing a more stringent result. Therefore, we defined the core genome as homologous genes shared by at least 97% of the genomes (^TOT^core_97_), which produced a core genome of 2663 clusters, representing only 1.96% of the total pangenome clusters. The ^TOT^core_97,_ colored green in Fig. 3a, contains the well-preserved genes that define the species, and for the shortest sequenced genomes (e.g. *E. coli* str. K-12 substr. MDS42, phylogroup A), these constitute ~74% of their gene content; in contrast, for the largest genomes (e.g., *E. coli* Ec138B_L1, phylogroup A), this fraction is only about 32%.

**Table 1:** Summary of pangenome analysis results.

Phylogroup	Core genome (97% strains)		Accessory genome		Unique		Total (Pangenome)		Core/pan (%)	No. of strains
	Clusters	Proteins	Clusters	Proteins	Clusters	Proteins	Clusters	Proteins	Clusters	
All	2663	28,566,052	82,821	22,783,754	50,499	51,099	135,983	51,400,905	1.96	10,667
A	3184	7,142,893	41,769	3,246,591	24,501	24,828	69,454	10,414,312	4.58	2232
B1	3141	9,365,646	44,019	4,887,086	24,590	24,844	71,750	14,277,576	4.38	2960
B2-1	3708	2,016,812	10,990	619,867	7048	7180	21,746	2,643,859	17.05	541
B2-2	3425	4,709,983	22,762	1,819,538	12,566	12,763	38,753	6,542,284	8.84	1367
C	3899	2,132,258	10,413	738,879	5242	5290	19,554	2,876,427	19.94	540
D1	3666	1,006,271	10,012	318,372	7659	7770	21,337	1,332,413	17.18	273
D2	3524	626,693	11,703	221,033	6765	7181	21,992	854,907	16.02	177
D3	3754	668,359	7252	201,292	4814	4936	15,820	874,587	23.73	177
E1	3151	885,018	14,883	471,354	7969	8088	26,003	1,364,460	12.12	279
E2(O157)	4060	3,080,073	6128	743,413	4442	4535	14,630	3,828,021	27.75	750
F	3486	698,031	9465	288,420	5381	5480	18,332	991,931	19.02	199
G	3783	365,756	5716	98,269	4016	4066	13,515	468,091	27.99	96
Shig1	3128	564,868	4903	256,426	2815	2883	10,846	824,177	28.84	177
Shig2	3732	3,383,814	6870	719,247	4751	4799	15,353	4,107,860	24.31	899

Values obtained from the different pangenome analysis using the 14 phylogroups separately and the entire set of assembled genomes (10,667 genomes) using UCLUST^47^. The same parameters were used throughout all of the analysis.

In the same manner, we defined the specific core genome for each of the phylogroups of the study (^PHY^core_97_) using the same parameters described above. By defining these phylogroup-specific core genes, it becomes apparent that large differences exist between the levels of gene preservation for each of the phylogroups (Fig. 3a). Predictably, the phylogroup with the largest number of ^PHY^core_97_ gene clusters is E2(O157). Not only do its members have large genomes, but this phylogroup is also very homogeneous as it mostly contains *E. coli* O157:H7 strains that have a clonal origin^30^. Relatively large ^PHY^core_97_ are also observed for phylogroups C, harboring strains of clinically relevant non-O157 enterohemorrhagic (EHEC) serotypes such as O111 and O26, and for phylogroup Shig2. The latter contains relatively short genomes and it is mainly composed of *Shigella sonnei* strains, suggesting that these phylogroups are relatively homogeneous which increases the size of the core genome and decreases the fraction of accessory genes. At the other end of the spectrum, the phylogroup with the smallest core genome is Shig1 followed by phylogroups B1, E1, and A (Table 1). The small core genome of Shig1 is related to its small genome size, whereas more numerous phylogroups A, E1, and B1 contain more diverse members, resulting in a larger fraction of accessory genes and a smaller phylogroup-specific core. This observation concurs with the tendency of environmental species that usually present open pangenomes with higher ratios of accessory and unique genes^31,32^. Nevertheless, although Shig1 phylogroup has the smallest number of core genes, this number represents almost 29% of the total clusters found in this phylogroup (Table 1), which is the highest ratio of core gene clusters per phylogroup-specific pangenome of the analysis. Phylogroups with fewer members can also produce larger core genome fractions with respect to their pangenome due to sampling biases, as exemplified by phylogroup G. This phylogroup was recently described as a multidrug-resistant extra‐intestinal pathogenic (ExPEC) phylogroup^13^. G strains are closely related to strains from the B2 complex, and are commonly isolated from poultry and poultry meat products, which coincides with our analyses and available metadata. Although phylogroup G has the fewest number of strains in our dataset, we believe that the high core/pan ratio of this phylogroup is due to the overabundance of the sequence type ST117 (79% of the strains), which makes this phylogroup quite homogeneous. Based on these observations, we propose that although the relative ratio of ^PHY^core_97_ to the total phylogroup pangenome clusters is a measure of the intragroup diversity. This must be interpreted with care, due to the uneven representation of genomes in the dataset.

To analyze the distribution of the 14 phylogroups in terms of their shared genetic content, a two-dimensional projection of the presence or absence of all protein families (complete pangenome) for the 10,667 assembled genomes is represented by a Principal Coordinate Analysis (PCoA) as shown in Fig. 3b. Phylogroups segregating on the left side of the Y axis (B2-1, B2-2, G, F, D1, D2, D3) comprise phylogroups that contain large numbers of strains labeled as extra-intestinal *E. coli* strains (ExPEC)^13,15,33^. The observed overlap of B2-1 with the B2-2 phylogroup in Fig. 3b could be due to their shared evolutionary history. For example, in silico MLST analyses shows that at least 80% of B2-1 strains belong to the sequence type ST131, a multidrug-resistant clonal complex of ExPEC strains that recently emerged from the B2-2 phylogroup^34^. This explains the high degree of homogeneity of the B2-1 phylogroup. Moreover, strains characterized as ST131 were not found in other phylogroups in our dataset. It appears that the rapid and differential acquisition of unique virulence and mobile genetic elements by ST131 strains^35^ allows discrimination between B2-1 (mainly ST131 strains) and B2-2 phylogroups using WGS approaches such as applied here.

Although most of the phylogroups seem to have a relatively horizontal distribution within the PCoA plot, phylogroups E2(O157) and Shig2 show a striking variance in their vertical distribution compared to the other phylogroups. As commented above, Shig2 and E2(O157) are relatively homogeneous phylogroups, with a large ^PHY^core_97_ that contains more than 1000 protein families than the ^TOT^core_97_ of the species. These phylogroup-specific core genes may contain genetic signatures that are not present in the core genome of the other phylogroups, resulting in intrinsic and distinguishable traits that are detectable in the PCoA analysis, and that differentiate them in terms of genetic content from the rest of the phylogroups.

To represent the existence of unique phylogroup-specific core genes, we made a comparison only considering the 14 ^PHY^core_97_ genes. These were re-clustered using the same parameters as in the previous pangenome analyses. Figure 3c is a representation of the sorted resultant clusters, placing clusters from the ^TOT^core_97_ first, followed by the ^PHY^core_97_ clusters from the rest of phylogroups. Sorting the clusters in this way highlights clusters of core genes that are exclusive to the ^PHY^core_97_ of a given phylogroup. As can be observed, phylogroups E2(O157) and Shig2 possess the highest proportion of unique core genes (protein family clusters (columns) colored in purple that are not present in the other phylogroups), followed by C, B2-1, and Shig1 phylogroups. Well-defined phylogroup unique core genes were also found for D3 (often containing uropathogenic multidrug-resistant strains, mainly ST405 and ST38) and D1 (containing uropathogenic multidrug-resistant strains, predominantly ST69). These phylogroup-specific core genes, along with their associated functional features, are listed in Supplementary Data 4. Some of these gene clusters comprise interesting characteristics, of which a limited selection is worth briefly mentioning here. A unique set of genes for synthesis of flagella is present in all strains belonging to the C phylogroup, which differs from flagellar synthesis genes in the other phylotypes; a complete set of genes for the transport of iron and ribose is present in all members of phylogroup E2(O157) but variably present in other phylotypes; and a set of genes for the synthesis of siderophores is found conserved in B2-1 phylogroup members (Supplementary Data 4). These mentioned genes may occasionally be observed in other phylogroups but are part of the ^PHY^core_97_ for the specified phylogroup only. These observations support the existence of 14 distinguishable phylogroups within the species that have different gene contents, although these genes result in phenotypes (motility, iron uptake, etc.), which are found in most members of all phylogroups. Thus, the phylogroups, originally identified by MLEE^36^ and here redefined by MASH analysis, are distinguishable also by gene content, for traits that are nevertheless phenotypically widespread within the species.

However, not all recognized phylogroups harbor clearly recognizable phylogroup-specific core genes. Phylogroups A and B1 have the weakest unique core signatures observed (along with D2 and E1 phylogroups), which could be explained by the heterogeneous nature of both phylogroups. Although B1 comprised strains mainly isolated from fecal sources, it also contains enteropathogenic strains (EPEC), EIEC strains, and most of the *Shigella* strains, such as *Shigella* *boydii* and *Shigella* *dysenteriae*, which were not classified by Mash analysis as Shig1 or Shig2 phylogroups (Supplementary Fig. 1 and Microreact data). These *Shigella* strains within B1 form a small group in the PCoA plot as the B1 small cluster just on top of the Shig1 cluster. It is interesting to note that, although phylogroups A and B1 are well-defined and historically described phylogroups, they are also considered as a sister phylogroups with a shared evolutionary history^9,15,37^. This is corroborated by our analysis, e.g., by their partial overlap observed in Fig. 3b and their late segregation observed in the movie available via figshare (10.6084/m9.figshare.13105235) and Fig. 2b at a Mash distance of 0.0115.

### Phylogroups evolve with different gain/loss rates of protein families

**Fig. 4:**
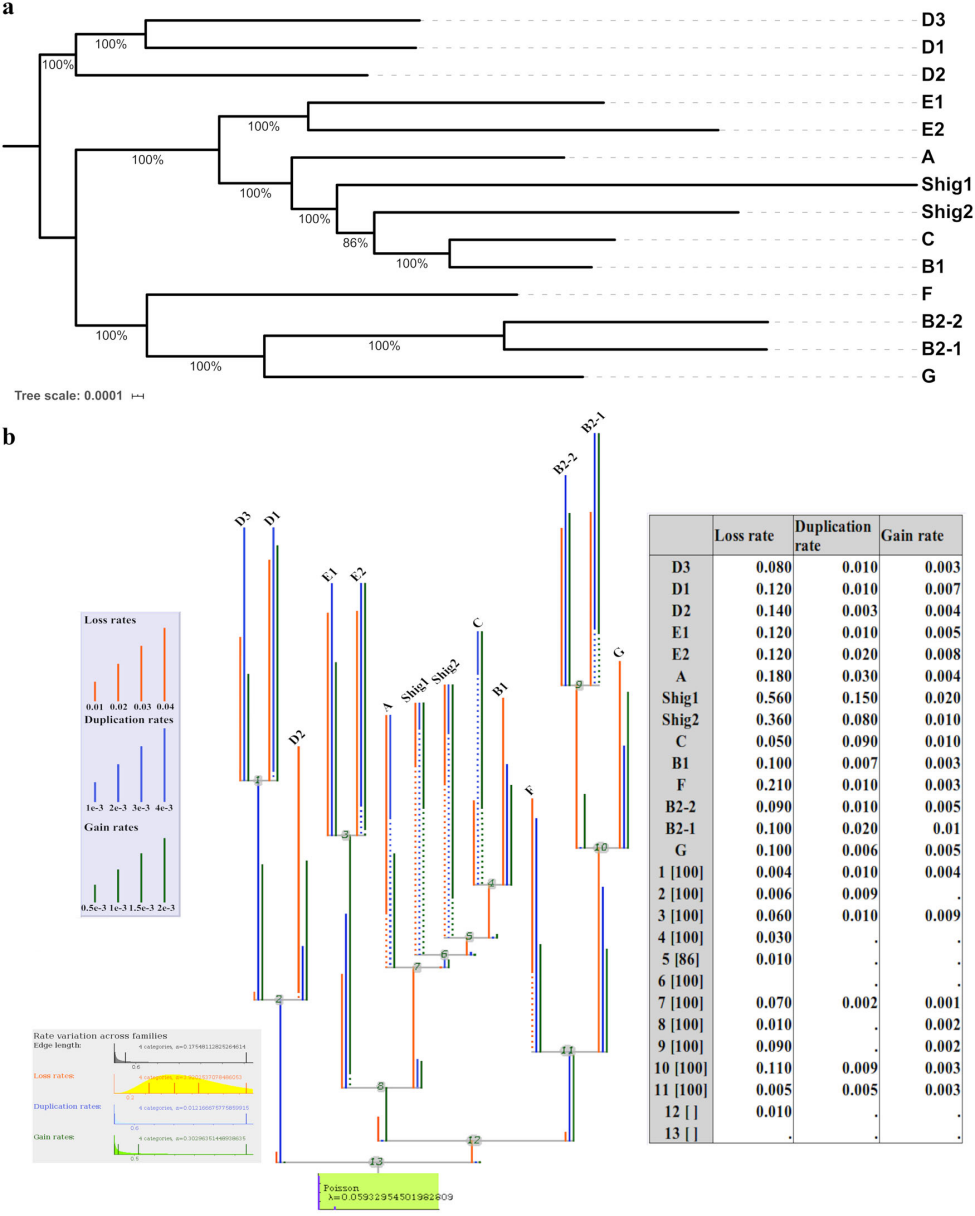
Phylogenetic representations of *E. coli* species using the core genome of the 14 medoids. **a** The tree was built using a set of 2,613 core clusters with no paralogs using IQ-TREE^50^. **b** Summary representation of Count output. The phylogenetic tree presents the different gain/loss/duplication ratios obtained per each phylogroup as output of Count v.10.04 software^51^. Dots in branches represent “informative ellipsis” where the length of the undotted section of the branch multiplied by the inverse ratio of undotted section is equal to the true rate of the branch. For example, assuming the displayed branch length is 1 and 1/10 of the branch is solid, then the true rate of the branch would be 10. Gain/loss/duplication rates for each branch are shown in the table.

As the medoids were shown to be suitable representative entities of the 14 phylogroups and the ^TOT^core_97_ genome was established, a robust phylogeny analysis could now be performed based on the concatenated independent alignment of 2613 ^TOT^core_97_ gene clusters without paralogs using a maximum likelihood approach (Fig. 4a). Such an approach would appear to be difficult, if not almost impossible, with datasets of more than a thousand genomes, due to computational costs. The obtained phylogenetic tree, along with a matrix containing the number of homologous genes per protein family for each representative genome, were used to measure family sizes and lineage-specific events applying an optimized gain–loss-duplicated model. Differences in gene content between the medoids lead to the observation that the different phylogroups have evolved with different gain/loss/duplication rates of protein families (Fig. 4b). Relatively high ratios of gene expansion were observed for phylogroups Shig1, Shig2, C, and B2-1. As expected, due to their smaller genomes, Shig1 and Shig2 possess the highest ratios of gene loss, while Shig1, C, and Shig2 have the highest rates of gene duplication. On the other hand, phylogroups A, B1, D2, D3, and F have the lowest rates of gene gain, indicating these phylogroups have undergone limited gene expansion. It is also interesting to note that phylogroups D2, B1, and G have much lower rates of gene duplication compared to the other phylogroups. In short, all phylogroups showed differential gain/loss duplication ratios of gene families, even those that share a presumed ancestral history, such as the B2, D, and E phylogroups. Interestingly, differences observed in genome size between members of phylogroups E1 and E2(O157) (Supplementary Fig. 17) seems to be due to a higher ratio of duplication relative to the acquisition of new gene families. High ratios of gene duplication in EHEC strains and other *E. coli* pathotypes has been reported in other studies^38^. As stated before, D1 and D3 phylogroups contain many UPEC strains that are mainly represented by one or two predominant sequence types. Conversely, D2 strains are typically isolated from non-human sources with a large variation of sequence types. Differences in gain/loss/duplication ratios are indeed more evident in D2 strains with a remarkable ratio of gene loss regarding the other D phylogroups.

## Discussion

Mash-based analysis provides a fast and highly scalable k-mer based approach that can be used on extremely large sets of genomes^21^. The methodology applied here enabled us to classify a cleaned set of 10,667 assembled *E. coli* strains (Fig. 1) and a filtered set of 95,525 unassembled genomes (Fig. 2c). Based on the analysis of more than one hundred thousand genomes, the population structure of *E. coli* species appears to be more diverse than currently thought^19,24^. The described Mash-based clustering method detected 14 phylogroups with a remarkably unequal distribution of membership in regards to the number of genomes per phylogroup, unveiling a bias in raw sequencing data towards members of phylogroups A, C, B1, and E2(O157). A different bias was observed for assembled genomes with B1, A, B2-2, and Shig2 being the most populated phylogroups.

Findings based on Mash analysis were supported by differences found in the genomic content of the 14 phylogroups that can be broadly defined into two categories: differences in conserved genes between and within phylogroups and differences in their phylogenetic profiles. Comparison of the pangenomic profiles via PCoA analysis revealed that each of the phylogroups possess a unique distribution within the space with the exception of phylogroups that share a recent evolutionary history such as phylogroups B2-1 and B2-2, or A and B1 (Fig. 3b). Moreover, the 14 phylogroups presented different levels of preservation of their core genome (Fig. 3a) and different gain/loss rates of protein families (Fig. 4b). It is also worth mentioning that most of the phylogroups described here contain unique core genes shared by all the strains of that phylogroup, but which are not present in the core genome of the others (Fig. 3c).

Most of the phylogroups identified here that have not previously been described consist of strains grouped in mainly one or more clonal complexes, such as ST131 in the case of B2-1 phylogroup and O157:H7 in the case of E2(O157). Although it is widely accepted that the genetic structure of *E. coli* species is predominantly clonal^10,39^, recent analyses have acknowledged that recombination events between *E. coli* strains occur at almost the same frequency as mutations; nevertheless, the ratio and scale of recombination is not sufficient to avoid the propagation of clonal lineages^40^. Further studies have suggested that the frequencies of homologous recombination and mutation rates in *E. coli* species can vary considerably and may correlate mainly with the lifestyle of the investigated isolates. Both kinds of mutational events are higher in pathogenic than in commensal members of the species and positively correlate with the number of virulence genes of each strain^40,41^ and with physiological conditions related to the environment^42^. The ratios of core/pangenome from Table 1 show that the core genome of phylogroups dominated by pathogenic and multidrug-resistant strains is significantly more preserved than in those phylogroups characterized by the presence of commensal (B1) and engineered and lab strains (A). This is in accordance with previous works that claim that recombination events seem to occurs more frequently in the core genome of the commensals strains and are less provable in the core genome of intestinal pathogens and multidrug-resistant extra-intestinal pathogenic strains^43^, which accumulate increasing levels of variability in their accessory and unique genomes.

A comparison of our classification method to Oliver Clermont’s accepted PCR phylogrouping protocol (as implemented in ClermonTyping, which mimics in vitro PCR assays^24^) revealed that 9% of the genomes in our dataset are untypable by in silico PCR-based methods. In addition, another 7% of the genomes had discrepancies between ClermonTyping’s PCR-based prediction and the Mash screen-based prediction implemented in the ClermonTyping method. Strikingly, ClermonTyping predictions conflicted for 47 of the 96 phylogroup G genomes in this study; PCR-based predictions indicated these strains were in phylogroup G but the Mash screen-based prediction indicated that these strains belong to phylogroup F.

The exponential increase in data availability requires new computational methods utilizing meaningful and balanced reductions to keep pace with the rate of data generation. For this, we suggest the use of medoids as representative entities for their respective phylogroup, the validity of which was demonstrated here. By utilizing all the assembled genomes to obtain an estimate of the population structure of *E. coli*, as currently sequenced, and reducing the population structure to a single representative for each subgroup, the computational demand of classifying unassembled reads (meaning the number of comparisons required) was reduced 762-fold. A reduction of this magnitude can enable researchers without high-performance computing resources to perform very large-scale population structure analyses that previously would have been computationally impractical. In addition, utilizing a reduction methodology similar to the one described here to reduce the population structure of a bacterial species to a 1 : 1 ratio among all subgroups helps reduce the effects of sequencing bias by ensuring each subgroup has equal representation in any subsequent analyses using the reduced set of genomes.

The results presented here indicate that the use of WGS analytical techniques, such as Mash, enable the detection of traceable genomic changes that are not detected by more target-restricted methods such as PCR. The use of WGS analysis techniques also can enable researchers to rapidly detect the emergence and spread of new pathogenic and/or antibiotic-resistant clonal complexes. We therefore conclude that analysis of WGS data using Mash to assess a bacterial species’ genetic substructure is a useful approach to increase our understanding of bacterial diversity.

## Methods

### Data acquisition and cleaning

To conduct the analysis, 12,602 genome sequences labeled either *Escherichia* or *Shigella* were downloaded from GenBank on 26 June 2018 using batch Entrez and the list of GCAs accession numbers from NCBI Genome database (including plasmid sequences when applicable). This dataset (Supplementary Data 1) was cleaned to obtain an informative and diverse set of 10,667 *E. coli* and *Shigella* genomes that captures the diversity of the species as sequenced to date. In addition to the GenBank genomes, a total of 125,771 read sets labeled as either *E. coli* or *Shigella* were downloaded from the SRA database. After cleaning the dataset, we utilized Mash^21^, a program that approximates similarity between two genomes in nucleotide content, and an in-house Python script to create a matrix of distances for all 10,667 genomes. This matrix was then clustered using hierarchical clustering after converting the Mash distance to a Pearson’s correlation coefficient distance, to ensure that clustering results were based on a genome’s overall similarity to the whole species.

To evaluate the quality of the dataset, various sequence quality scores were calculated as described by Land et al.^44^. Following the recommended quality score cutoff value of 0.8, the dataset was filtered to include only genomes with a total quality score of 0.8 or higher. Applying the same cutoff value to the sequence quality score alone resulted in an extremely restricted dataset that no longer addressed the goals of this study. Genome size was restricted to >3 Mb and <6.77 Mb, to remove questionably sized genomes, which could be due to contamination or modified genomes that are not representative of the natural *E. coli* species. After applying these two steps, 10,855 genomes remained in the assembled genome dataset for analysis.

To further clean the dataset, we filtered genomes that were outside the statistical distribution of Mash distances within the dataset. Assuming that *Shigella* species are all members of *E. coli*, we decided to use type strains for the *Escherichia* and *Shigella* genera (accession numbers GCA_000613265.1 and GCA_002949675.1, respectively) to quickly filter the set of 10,855 genomes for erroneous or low-quality genomes that may have slipped through the previous cleaning steps. The Mash values of the 10,855 genomes compared to each type strain were broken into percentiles ranging from 10% to 99.995%. A cutoff percentile of 98.5% was determined to provide sufficient cleaning without risking a large loss of data (Supplementary Data 1) and was applied to each type strain Mash value set. Genomes that were found in both sets after filtering were retained to produce the final dataset of 10,667 genomes.

### Microreact analysis

Microreact^22^ was utilized to visualize the resultant clustering of the Mash data, as this provides an easy and fast medium to further explore the results of the analysis. To leverage the search capabilities of Microreact, we mapped metadata found for our dataset from the database PATRIC^23^ (downloaded on 20 June 2019). This allows the exploration of our results using a number of shared characteristics and queries such as “geographic location” or “serovar,” which, although outside the scope of the current study, could be used as a topic for future analyses to increase our understanding of *E. coli* species. In addition, to enable easy comparison between established phylogroup predicting methods, we have provided columns containing the results of the ClermonTyping^24^ method on our dataset.

### Mash and clustering analysis

Genetic distances between all 10,667 genomes were calculated using “mash dist” with a k-mer size of 21 and a sampling size of 10,000. The resulting output was converted into a distance matrix with assembly accession numbers as columns and rows. To improve the clustering results and to provide a standard metric that allows comparison of different analytical methods, we converted the Mash distance value into a similarity measure via the Pearson’s correlation coefficient^45^. This returns values ranging from −1 (total negative linear correlation) to 1 (total positive linear correlation), where 0 is no linear correlation. As clustering-based methods require a distance measure, the values were subtracted from 1 to convert them into a distance measure. These distance measures were then clustered in R using “hclust” and the “ward.D2” method. A clustered heatmap was generated using the hclust dendrogram to reorder the rows and columns of the distance matrix within the heatmap, whereas values from the raw distance matrix of Mash distances were mapped to color. To determine the height to cut the hclust dendrogram and to accurately predict phylogroups that optimally overlapped with existing phylogroups, we compared multiple different cutoff values and methods to obtain cutoff values. Taking the maximum height present in the hclust dendrogram and multiplying it by 1.25 × 10^−2^ was found to provide both accurate predictions and a standard method that scales with the data supplied. The final phylogroups were detected using a cut-off that split phylogroup C from phylogroup B1. This implies that the 12 remaining phylogroups exist at a higher level of genetic difference than the B1/C phylogroups. Therefore, our methodology assumes that C phylogroup is a true phylogroup rather than a specific genetic lineage of the B1 phylogroup, as it has been previously described by Clermont quadruplex PCR methodology^11^. Some detailed results of both the cutoff percentile and hclust height testing are included for 10,667 genomes in Supplementary Data 1.

### Medoid selection for species representation

Using the Mash values for the entire species, a medoid was defined for each phylogroup. The medoid is the “real” center of the phylogroup, as it has to exist within the dataset, and was chosen as the genome that has the lowest average distance to all other genomes in its phylogroup. We subsequently tested if one genome from each of the phylogroups would be enough to accurately classify any given genome sequence claimed to be *E. coli* or *Shigella*. The “aggregate” function of R was used to find the mean across each phylogroup. Isolating each phylogroup, reclustering, and calculating the medoid did not yield as accurate results as calculating the medoid per phylogroup with respect to the entire 10,667 genome dataset.

### Addition of SRA reads

The keywords “*Escherichia coli*” and “*Shigella*” filtered with “DNA” for biomolecule and “genome” for type was used to retrieve SRA IDs from the NCBI SRA database on 22 March 2019. For large-scale data transfer, these SRA genomes were downloaded using the high-throughput file transfer application Aspera (http://asperasoft.com). To ease computational and organizational load, the 125,771 read sets obtained from the SRA were divided into 5 subsets of different sequencing technologies: 3 Illumina paired read sets, 1 mixed technology with paired reads, and 1 mixed technology with single reads. The five sets of reads were then converted from fastq to fasta format, to be processed by Mash using a python script, which removed all non-sequence data from the fastq file.

The SRA sequence reads were sketched using Mash (v2.1) and the same k-mer and sketch sample size as the 10,667 dataset. This version change was due to the addition of read pooling in the read mode which automatically joins paired reads, eliminating the need to concatenate or otherwise process paired read sets. All read sets were sketched individually so that read sets that caused an error when sketching were dropped from the analysis before sketching. A total of 23,680 raw reads could not be sketched. The -m setting was set to 2 to decrease noise in the sketches of the reads. After sketching the reads within the subsets, all sketches were concatenated into a sketch for that subset using the paste command of Mash. The concatenated sketch of each subset was then compared to the 14 medoids using Mash dist. As all five subsets had the same reference, the distance output from each subset was concatenated to one file. This single SRA distance output file was then analyzed to evaluate the quality of the SRA dataset. Due to how distances are calculated, Mash can consistently flag genomes of very low quality, as the major basis of a Mash value is how many hits are present out of sketches sampled. The top 5 most numerous distances of the SRA read sets corresponded to 0–4 hits of the possible 10,000 sketches per genome. This indicates the presence of extremely low-quality samples within the SRA dataset. A histogram of the SRA Mash distance results was created to analyze the distribution of Mash distances of the entire 102,091 SRA dataset (results not shown). A final Mash distance cutoff of 0.04 was chosen based on the maximum Mash value in the 10,667 whole set that was 0.0393524. Although this low cutoff might potentially eliminate useful information, it insured quality of the SRA dataset. This retained 95,525 reads that had at least one Mash distance to a phylogroup medoid within the chosen cutoff.

The distance output was transferred into a matrix with reads as columns and rows containing a phylogroup medoid. For each read, the smallest Mash distance to a medoid was identified, and the corresponding medoid noted (Supplementary Data 2). We then created a distance matrix from the Mash distance output of the 95,525 reads that met the above cutoff with reads as rows and medoids as columns. Due to computational load this distance matrix was loaded into Python 3 instead of R. A clustered heatmap was made using Seaborn, Matplotlib, and Scipy with the “clustermap” function. Instead of clustering both rows and columns, columns (phylogroups) were ordered the same as Fig. 1 and rows were sorted as follows: number of hits to phylogroups (ascending = True) and Mash distance (ascending = False). This provided a quick visualization method for the SRA dataset with a consistent sorting criterion to make comparison between Fig. 2c and the supplemental heatmaps (Supplementary Figs. 3–16) much easier.

### Cytoscape visualization

The Mash distance matrix of the 10,667 genomes was filtered to include only the 14 medoids along the columns. This filtered matrix was transformed into a new three column matrix, where the first column contains the identifier for a genome to be compared to the medoid present in the second column. The third column contains the Mash value for that pairwise comparison. A sliding cutoff ranging from 0.04 to 0.0095 with increments of 0.005 was applied to the Mash value column and rows with values above the sliding cutoff for an iteration were removed. These data tables were imported into Cytoscape (version 3.7.1) with the first column as the source node and the medoid column as the target node. The Prefuse Force Directed Weighted layout was then applied to the network with the Mash distance serving as the weight. Phylogroup membership was mapped with a metadata table and colors were assigned based on the colors used in Fig. 1. For each cutoff, the resultant graph was output as a scalable vector graphic (SVG) image. All SVGs were then compiled into a video to ease visualization of the Cytoscape graphs.

### Statistical analysis of genome sizes and percent GC content

Genome sizes and percent of GC content was calculated using the “infoseq” package from EMBOSS suite v6.6.0.0. A data frame with sequence ID, percentage of GC content, genome size, and phylogroup ID was made. Library “ggplot2” from R was used to plot genome sizes and GC content. Library “dplyr” from R was used to perform ANOVA test and Tukey’s honest significant difference (HSD) tests. The homogeneity of variances was tested using Levene’s test and the normality assumption of the data were checked using Shapiro–Wilk test. As some of the groups did not meet the criteria of the assumption of normality, Kruskal–Wallis test was performed and non-parametric alternative to one-way ANOVA as well. Kruskal–Wallis test rejected both null hypothesis (means of genome size or percent of GC content are similar between the different phylogroups), with *p*-value < 2.2e^−16^ in both cases. Raw results from these tests are available in Supplementary Data 3.

### Pangenome analyses and clustering

All 10,667 genomes were reannotated using Prokka^46^ v1.13, with parameters: --rnammer --kingdom Bacteria --genus *Escherichia* –species *coli* --gcode 11. All protein-coding sequences (*n* = 51,400,905) were clustered using UCLUST from USEARCH^47^ v.10.0.240 into protein families using cutoff values of 80% of protein sequence similarity, 80% of query sequence coverage, *e*-value ≤ 0.0001 (parameters -evalue 0.0001 -id 0.8 -query_cov 0.8, with maxaccepts 1 and maxrejects 8). For the core genome, various inclusion percentages were compared, as we included draft genomes existing in multiple contigs. The optimum was defined that allowed 3% omissions, giving a species core genome defined as those genes present in 97% of the genome collection. Therefore, protein families with presence in at least 97% of the total set strains were considered part of the core genome of *E. coli* species.

The pan- and core genome for each of the 14 phylogroups were then separately clustered using the same cut-off parameters as the entire set at species level.

### MLST analysis

The sequence type for all 10,667 assembled genomes was assessed using the program “mlst” version 2.18.0 from Seemann T, Github: https://github.com/tseemann/mlst, using both the Achtman and Pasteur MLST schemas for *E. coli* from PubMLST website (https://pubmlst.org/) developed^48^ by Keith Jolley. Results were collected and are accessible in our microreact database: https://microreact.org/project/10667ecoli/b4431cf8.

### Core genome matrix creation and visualization

Core genome clusters for the 14 phylogroups obtained using UCLUST v.10.0.240 in the previous analysis were used again with UCLUST v.10.0.240 using the same parameters to find the intersection of core genes between the core clusters of the 14 phylogroups. A binary matrix with cluster ID as column labels, genome IDs as row names, and the number of genes belonging to that cluster as the cell value was constructed using the main output from UCLUST. This matrix was then supplied to an “in-house” python script that sorts the pangenome matrix such that the gene clusters found in all phylogroups are placed first (species’ core genome). Then groups are sorted by abundance per phylogroup to isolate phylogroup core genes. All leftover gene groups are sorted by phylogroup and abundance, and added to the end of the sorted gene cluster list. The Mash tree obtained earlier for the 10,667 dataset was then loaded and used to sort the order of the organisms within the sorted matrix. Finally, Matplotlib was used to visualize the sorted matrix.

### Phylogenetic analysis of core gene families

The set of core gene clusters of the 14 medoids was extracted from the core genome clusters of the entire species and from them single copy ortholog groups were identified to construct a phylogenomic tree. In total a set of 2,613 single gene (clusters without paralogs paralogs) ortholog groups were aligned using MAFFT^49^ v.7.110. The model of evolution for each of the 2,613 protein clusters was calculated using IQ-TREE^50^ v.1.6.10 with parameters -m TESTONLY -nt AUTO. Once the best model of evolution was obtained for each of the core protein families, those clusters that shared model of evolution were sent together to IQ-TREE for a better estimation of the substitution model parameters using -m MF + MERGE, -nt AUTO and selecting the final model of evolution with mset parameter. In the last step, all partitions obtained with their corresponding model of evolution were sent again to IQ-TREE for final estimation of the phylogenetic tree for the 14 medoids using ultrafast bootstraping approach (-bb 1000). The resulted core genome tree was re-rooted using the B2-1, B2-2, and G phylogroups branch, according to the results obtained from the Mash analysis and the literature^19^.

The pangenome matrix needed as input for Count^51^ v10.04 for the 14 medoids was constructed using UCLUST (with same parameters for pangenome calculation as in previous analyses). A pivot table was built using the main output from UCLUST and pandas library in a Python 3 script using the function “pivot_table” with agglomeration function=sum. Count v10.04 program was used for gene family expansion/contraction analysis, using an optimized gain–loss-duplicated model^52,53^ using Poisson family size distribution, four gamma categories for each calculation across families (Edge length, Loss rate, Gain rate, and Duplication rate), and different lineage-specific variation for gain–loss ratio and duplication–loss ratio between lineages. Measurements were done using 1000 optimization rounds (reaching convergence before the last iteration) and 0.01 convergence threshold on the likelihood.

### Principal coordinate analysis

The PCoA plot in Fig. 3b was created using R, the entire pangenome matrix for the 10,667 assembled genomes, and the libraries “ade4” version 1.7-13 and “labdsv” version 2.0-1. A Jaccard distance matrix of the pangenome matrix was created using the ‘dist.binary’ function from “ade4.” To create the PCoA data, the Jaccard distance matrix was used in the ‘“pco” function of “labdsv” with *k* = 10,666 (allowing each genome to be a unique dimension). The resultant PCoA data were then graphically rendered using R “plot” and colors were added by genome classification as shown in Fig. 1.

### Statistics and reproducibility

Library “dplyr” from R was used to perform ANOVA test and Tukey’s HSD tests. The homogeneity of variances was tested using Levene’s test and the normality assumption of the data were checked using Shapiro–Wilk test. As some of the groups did not meet the criteria of the assumption of normality, Kruskal–Wallis test was performed and non-parametric alternative to one-way ANOVA as well. Kruskal–Wallis test rejected both null hypothesis (means of genome size or percent of GC content are similar between the different phylogroups), with *p*-value < 2.2e^−16^ in both cases. Raw results from these tests are available in Supplementary Data 3. Information regarding the genomes utilized in this study are available in Supplementary Data 1 (10,667 assembled genomes) and Supplementary Data 2 (95,525 unassembled genomes). The Mash sketches utilized in this study are available upon request.

### Reporting summary

Further information on research design is available in the Nature Research Reporting Summary linked to this article.

## Supplementary information

Supplementary Information

Description of Additional Supplementary Files

Supplementary Data 1

Supplementary Data 2

Supplementary Data 3

Supplementary Data 4

Reporting Summary

## Data Availability

The data supporting the findings of this study are available in this article, its Supplementary Information files, or on the Zenodo^54^ entry associated with this project. GenBank assembly accession codes for all assembled genomes used in this study can be found in Supplementary Data 1. Sequence Read Archive accessions for all unassembled genomes used in this study can be found in Supplementary Data 2. Source data for all manuscript figures can be found on Zenodo via 10.5281/zenodo.4091750. Each file in the Zenodo^54^ collection is labeled corresponding to the figure it is associated with. The code needed to reproduce all plots are also available on Zenodo^54^. It is noteworthy that the code in Zenodo^54^ replicates all parts of the analysis and thus the names may not be the same between the code and the underlying data. For each figure, the code that reproduces the figure is identified in the Description section of the Zenodo^54^ entry. An animated video with all the Cytoscape graphs is available on figshare via 10.6084/m9.figshare.13105235^55^. The images used as frames for the animated video are available on figshare via 10.6084/m9.figshare.11473308^56^.

## Code availability

Code for this study is available on Zenodo^54^ via 10.5281/zenodo.4091750. Software versions utilized in this study include the following: Mash v2.1, R v3.5.1, Python v3.6.8, Cytoscape v3.7.1, EMBOSS v6.6.0.0, USEARCH/UCLUST v10.0.240, MAFFT v7.110, IQ-TREE v1.6.10, and Count v10.04.

## References

[1] Jang J (2017). Environmental *Escherichia coli*: ecology and public health implications-a review. J. Appl. Microbiol..

[2] Alm, E. W., Walk, S. T. & Gordon, D. M. in *Population Genetics of Bacteria*. 69–89, 10.1128/9781555817114.ch6 (Wiley, 2011).

[3] Lan R, Reeves PR (2002). *Escherichia coli* in disguise: molecular origins of *Shigella*. Microbes Infect..

[4] Fischer Walker CL, Sack D, Black RE (2010). Etiology of diarrhea in older children, adolescents and adults: a systematic review. PLoS Negl. Trop. Dis..

[5] Dunne, K. A. et al. Sequencing a piece of history: complete genome sequence of the original *Escherichia coli* strain. *Microb. Genom*. **3**, mgen000106 (2017). 10.1099/mgen.0.000106 PMC5382810 28663823

[6] Pettengill, E. A., Pettengill, J. B. & Binet, R. Phylogenetic analyses of *Shigella* and Enteroinvasive *Escherichia coli* for the identification of molecular epidemiological markers: whole-genome comparative analysis does not support distinct genera designation. *Front. Microbiol*. **6**, 1573 (2016). 10.3389/fmicb.2015.01573 PMC4718091 26834722

[7] Chattaway MA, Schaefer U, Tewolde R, Dallman TJ, Jenkins C (2017). Identification of *Escherichia coli* and *Shigella* species from whole-genome sequences. J. Clin. Microbiol..

[8] Clermont O, Bonacorsi S, Bingen E (2000). Rapid and simple determination of the *Escherichia coli* phylogenetic group. Appl. Environ. Microbiol..

[9] Gordon DM, Clermont O, Tolley H, Denamur E (2008). Assigning *Escherichia coli* strains to phylogenetic groups: multi-locus sequence typing versus the PCR triplex method: MLST versus Clermont method. Environ. Microbiol..

[10] Tenaillon O, Skurnik D, Picard B, Denamur E (2010). The population genetics of commensal *Escherichia coli*. Nat. Rev. Microbiol..

[11] Clermont O, Christenson JK, Denamur E, Gordon DM (2013). The Clermont *Escherichia coli* phylo-typing method revisited: improvement of specificity and detection of new phylo-groups: a new *E. coli* phylo-typing method. Environ. Microbiol. Rep..

[12] Meier-Kolthoff JP (2014). Complete genome sequence of DSM 30083T, the type strain (U5/41T) of *Escherichia coli*, and a proposal for delineating subspecies in microbial taxonomy. Stand. Genom. Sci..

[13] Clermont O (2019). Characterization and rapid identification of phylogroup G in *Escherichia coli*, a lineage with high virulence and antibiotic resistance potential. Environ. Microbiol..

[14] Walk ST (2009). Cryptic lineages of the genus *Escherichia*. Appl. Environ. Microbiol..

[15] Carlos C (2010). *Escherichia coli* phylogenetic group determination and its application in the identification of the major animal source of fecal contamination. BMC Microbiol..

[16] Vangchhia B (2016). Phylogenetic diversity, antimicrobial susceptibility and virulence characteristics of phylogroup F *Escherichia coli* in Australia. Microbiology.

[17] Konstantinidis KT, Ramette A, Tiedje JM (2006). Toward a more robust assessment of intraspecies diversity, using fewer genetic markers. Appl. Environ. Microbiol..

[18] Sims GE, Kim S-H (2011). Whole-genome phylogeny of *Escherichia coli*/*Shigella* group by feature frequency profiles (FFPs). PNAS.

[19] Gonzalez-Alba, J. M., Baquero, F., Cantón, R. & Galán, J. C. Stratified reconstruction of ancestral *Escherichia coli* diversification. *BMC Genomics* **20**, 936 (2019). 10.1186/s12864-019-6346-1 PMC6896753 31805853

[20] Struyf, A., Hubert, M. & Rousseeuw, P. Clustering in an object-oriented environment. *J. Stat. Soft*. **1**, 10.18637/jss.v001.i04 (1997).

[21] Ondov BD (2016). Mash: fast genome and metagenome distance estimation using MinHash. Genome Biol..

[22] Argimón S (2016). Microreact: visualizing and sharing data for genomic epidemiology and phylogeography. Micro. Genom..

[23] Wattam AR (2017). Improvements to PATRIC, the all-bacterial Bioinformatics Database and Analysis Resource Center. Nucleic Acids Res..

[24] Beghain, J., Bridier-Nahmias, A., Le Nagard, H., Denamur, E. & Clermont, O. ClermonTyping: an easy-to-use and accurate in silico method for *Escherichia* genus strain phylotyping. *Microb. Genom*. **4**, e000192 (2018). 10.1099/mgen.0.000192 PMC6113867 29916797

[25] Zhou Z, Alikhan N-F, Mohamed K, Fan Y, Achtman M (2020). The EnteroBase user’s guide, with case studies on *Salmonella* transmissions, *Yersinia pestis* phylogeny, and *Escherichia* core genomic diversity. Genome Res.

[26] Shannon P (2003). Cytoscape: a software environment for integrated models of biomolecular interaction networks. Genome Res..

[27] Weinert LA, Welch JJ (2017). Why might bacterial pathogens have small genomes?. Trends Ecol. Evol..

[28] Bhunia, A. K. in *Foodborne Microbial Pathogens: Mechanisms and Pathogenesis* (ed. Bhunia, A. K.) 249–269, 10.1007/978-1-4939-7349-1_14 (Springer, New York, 2018).

[29] Balbi KJ, Rocha EPC, Feil EJ (2009). The temporal dynamics of slightly deleterious mutations in *Escherichia coli* and *Shigella* spp. Mol. Biol. Evol..

[30] Sharma VK, Akavaram S, Schaut RG, Bayles DO (2019). Comparative genomics reveals structural and functional features specific to the genome of a foodborne *Escherichia coli* O157:H7. BMC Genomics.

[31] Udaondo Z, Molina L, Segura A, Duque E, Ramos JL (2016). Analysis of the core genome and pangenome of *Pseudomonas putida*. Environ. Microbiol..

[32] Abreo E, Altier N (2019). Pangenome of *Serratia marcescens* strains from nosocomial and environmental origins reveals different populations and the links between them. Sci. Rep..

[33] Salipante SJ (2015). Large-scale genomic sequencing of extraintestinal pathogenic *Escherichia coli* strains. Genome Res..

[34] Nicolas-Chanoine M-H, Bertrand X, Madec J-Y (2014). *Escherichia coli* ST131, an intriguing clonal group. Clin. Microbiol. Rev..

[35] Petty NK (2014). Global dissemination of a multidrug resistant *Escherichia coli* clone. PNAS.

[36] Herzer PJ, Inouye S, Inouye M, Whittam TS (1990). Phylogenetic distribution of branched RNA-linked multicopy single-stranded DNA among natural isolates of *Escherichia coli*. J. Bacteriol..

[37] Lecointre G, Rachdi L, Darlu P, Denamur E (1998). *Escherichia coli* molecular phylogeny using the incongruence length difference test. Mol. Biol. Evol..

[38] Bernabeu M (2019). Gene duplications in the *E. coli* genome: common themes among pathotypes. BMC Genomics.

[39] Desjardins P, Picard B, Kaltenböck B, Elion J, Denamur E (1995). Sex in *Escherichia coli* does not disrupt the clonal structure of the population: evidence from random amplified polymorphic DNA and restriction-fragment-length polymorphism. J. Mol. Evol..

[40] Bobay L-M, Traverse CC, Ochman H (2015). Impermanence of bacterial clones. PNAS.

[41] Rodríguez-Beltrán J (2015). High recombinant frequency in extraintestinal pathogenic *Escherichia coli* strains. Mol. Biol. Evol..

[42] Aubron C (2012). Variation in endogenous oxidative stress in *Escherichia coli* natural isolates during growth in urine. BMC Microbiol.

[43] McNally A, Cheng L, Harris SR, Corander J (2013). The evolutionary path to extraintestinal pathogenic, drug-resistant *Escherichia coli* is marked by drastic reduction in detectable recombination within the core genome. Genome Biol. Evol..

[44] Land ML (2014). Quality scores for 32,000 genomes. Stand Genom. Sci..

[45] Kirch, W. (ed) in *Encyclopedia of Public Health* 1090–1091, 10.1007/978-1-4020-5614-7_2569 (Springer, The Netherlands, 2008).

[46] Seemann T (2014). Prokka: rapid prokaryotic genome annotation. Bioinformatics.

[47] Edgar RC (2010). Search and clustering orders of magnitude faster than BLAST. Bioinformatics.

[48] Jolley KA, Maiden MC (2010). BIGSdb: scalable analysis of bacterial genome variation at the population level. BMC Bioinformatics.

[49] Katoh K, Standley DM (2013). MAFFT multiple sequence alignment software version 7: improvements in performance and usability. Mol. Biol. Evol..

[50] Nguyen L-T, Schmidt HA, von Haeseler A, Minh BQ (2015). IQ-TREE: a fast and effective stochastic algorithm for estimating maximum-likelihood phylogenies. Mol. Biol. Evol..

[51] Csűrös M (2010). Count: evolutionary analysis of phylogenetic profiles with parsimony and likelihood. Bioinformatics.

[52] Csűrös M, Miklós I (2009). Streamlining and large ancestral genomes in Archaea inferred with a phylogenetic birth-and-death model. Mol. Biol. Evol..

[53] Olm MR (2017). Identical bacterial populations colonize premature infant gut, skin, and oral microbiomes and exhibit different in situ growth rates. Genome Res..

[54] Abram, K. et al. Mash-based analyses of *E. coli* genomes reveal 14 distinct phylogroups. *Zenodo*10.5281/zenodo.4091750 (2020). 10.1038/s42003-020-01626-5 PMC7838162 33500552

[55] Abram, K., et al. Supplementary Movie 1. figshare 10.6084/m9.figshare.13105235 (2020).

[56] Abram, K., et al. Supplementary Video 1 Stills. figshare 10.6084/m9.figshare.11473308 (2020).

