# Supplementary material for: Mash-based analyses of Escherichia coli genomes reveal 14 distinct phylogroups
Source: Commun Biol. 2021 Jan 26;4:117. doi: 10.1038/s42003-020-01626-5 (PMC7838162; doi:10.1038/s42003-020-01626-5)
Supplement: Supplementary file 1 — Supplementary Information [file 42003_2020_1626_MOESM1_ESM.pdf]

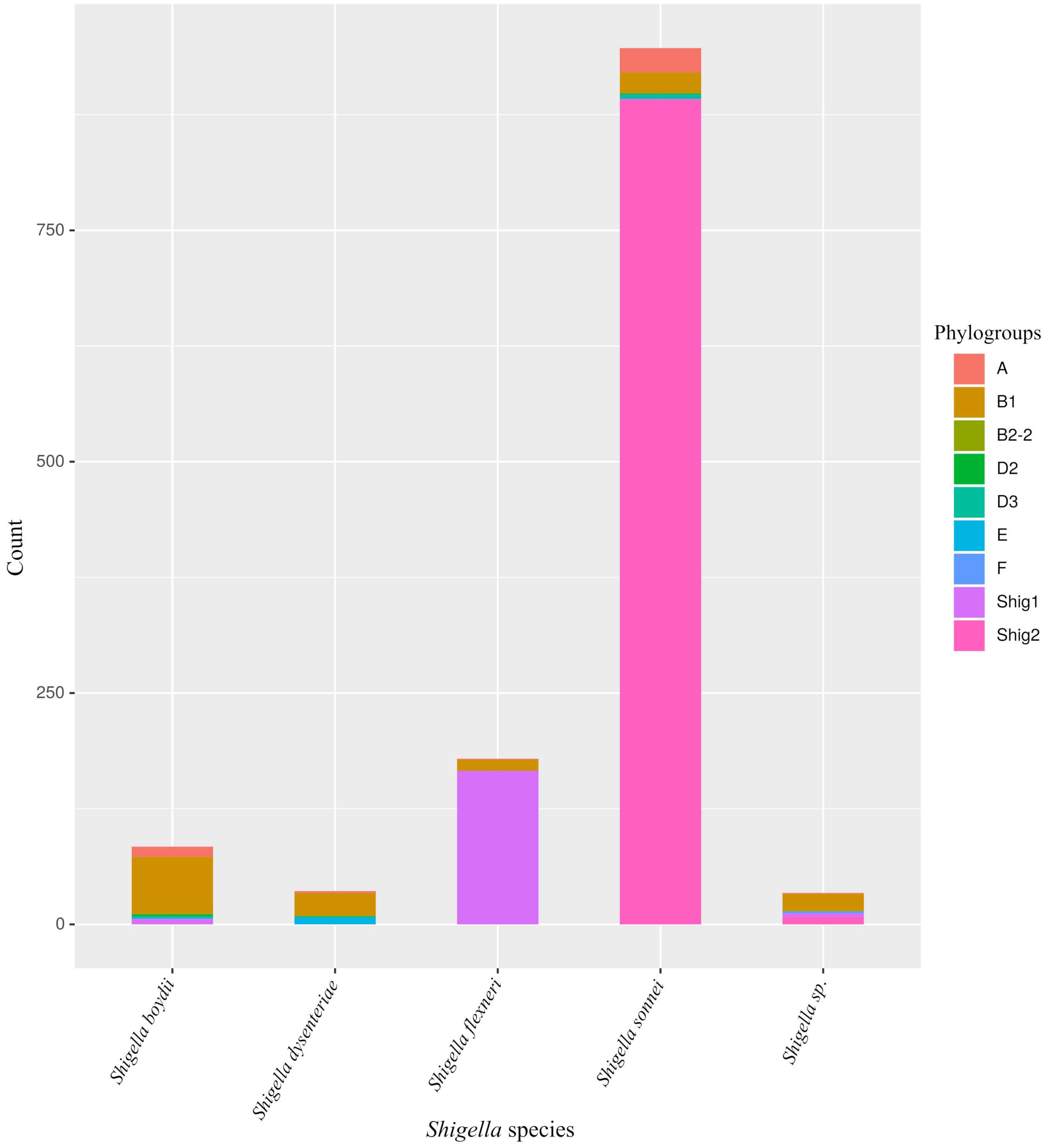

Supplementary Figure 1

Distribution of *Shigella* genomes over phylogroups

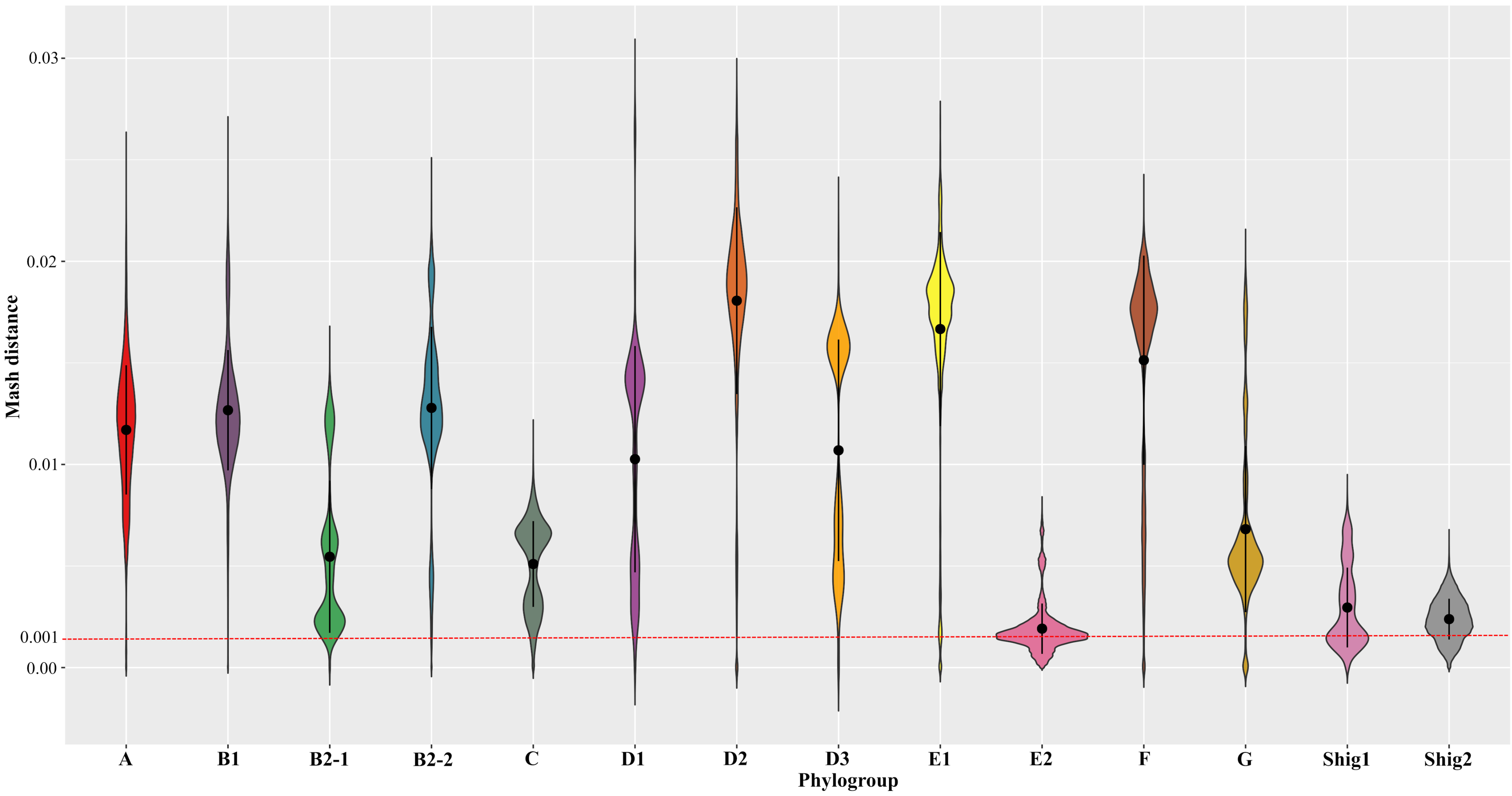

Supplementary Figure 2

Violin plots of the distribution of Mash distances per phylogroup. The red line corresponds to the Mash distance described as the cut-off for strain clonality according to the literature[53]

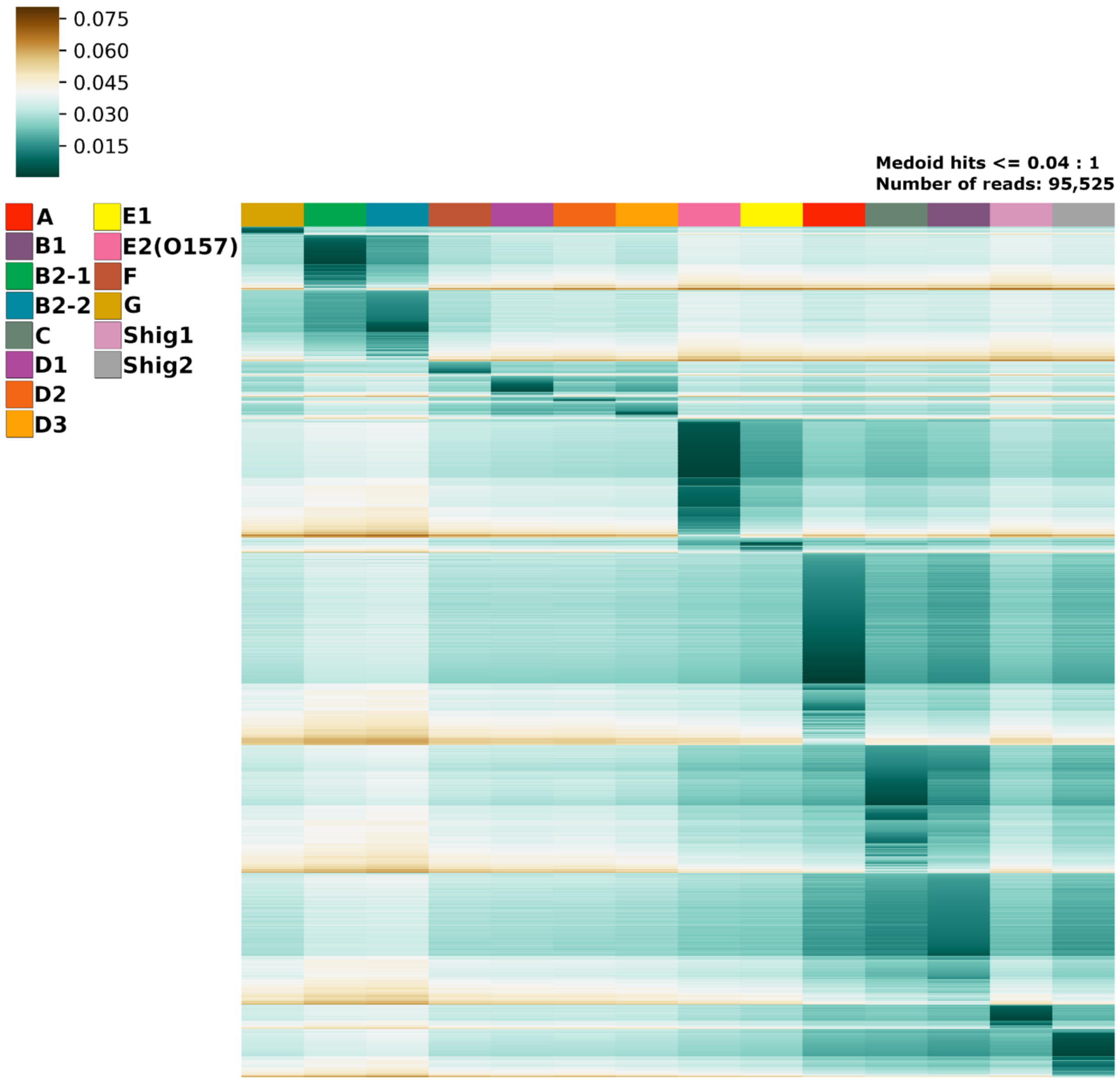

Supplementary Figure 3

Heatmap of Mash distances for 95,525 SRA reads to each of the 14 medoids. Reads were filtered to any read with a Mash distance ≤ 0.04 to at least 1 medoid.

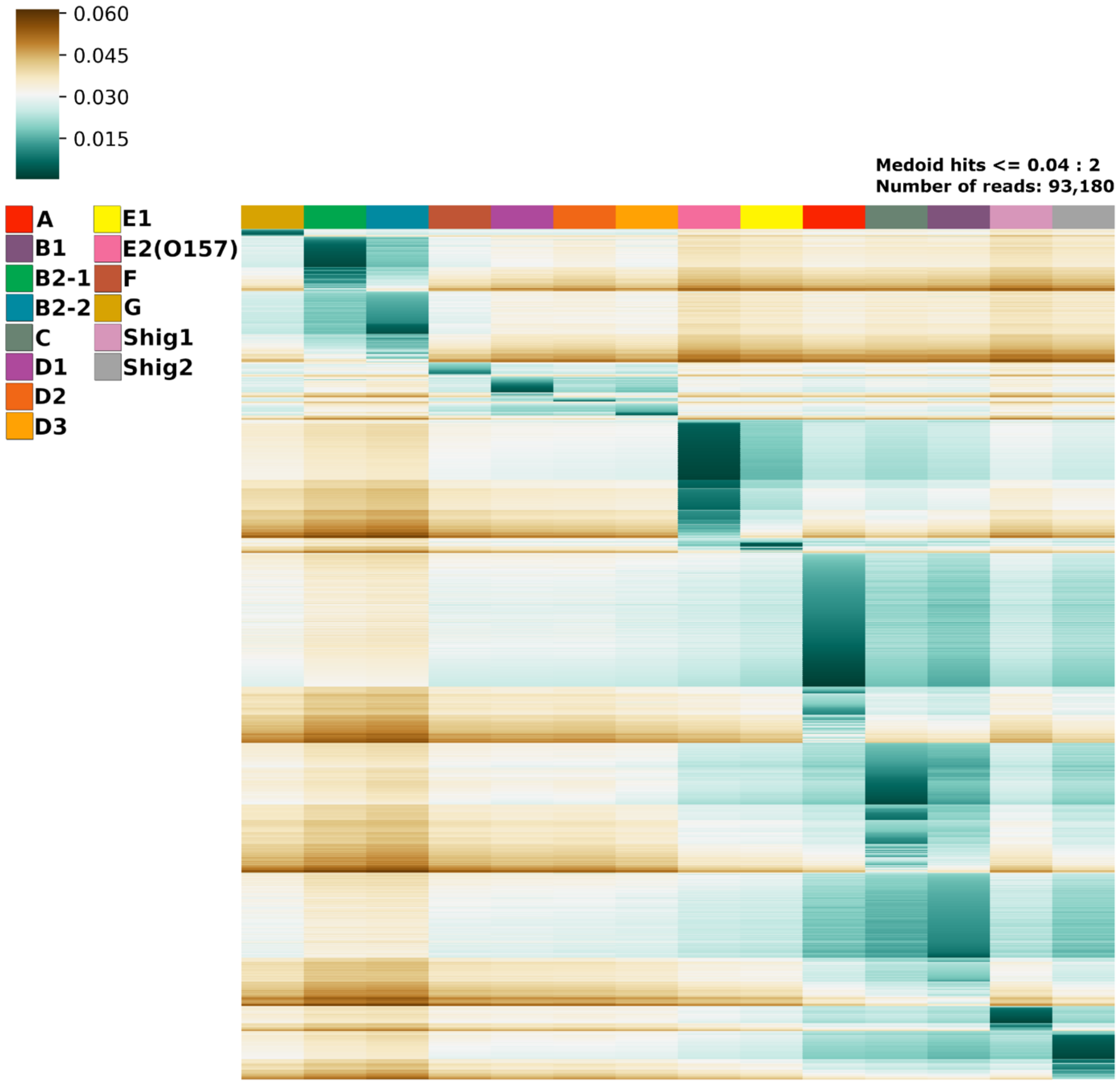

Supplementary Figure 4

Heatmap of Mash distances for 93,180 SRA reads to each of the 14 medoids. Reads were filtered to any read with a Mash distance ≤ 0.04 to at least 2 medoids.

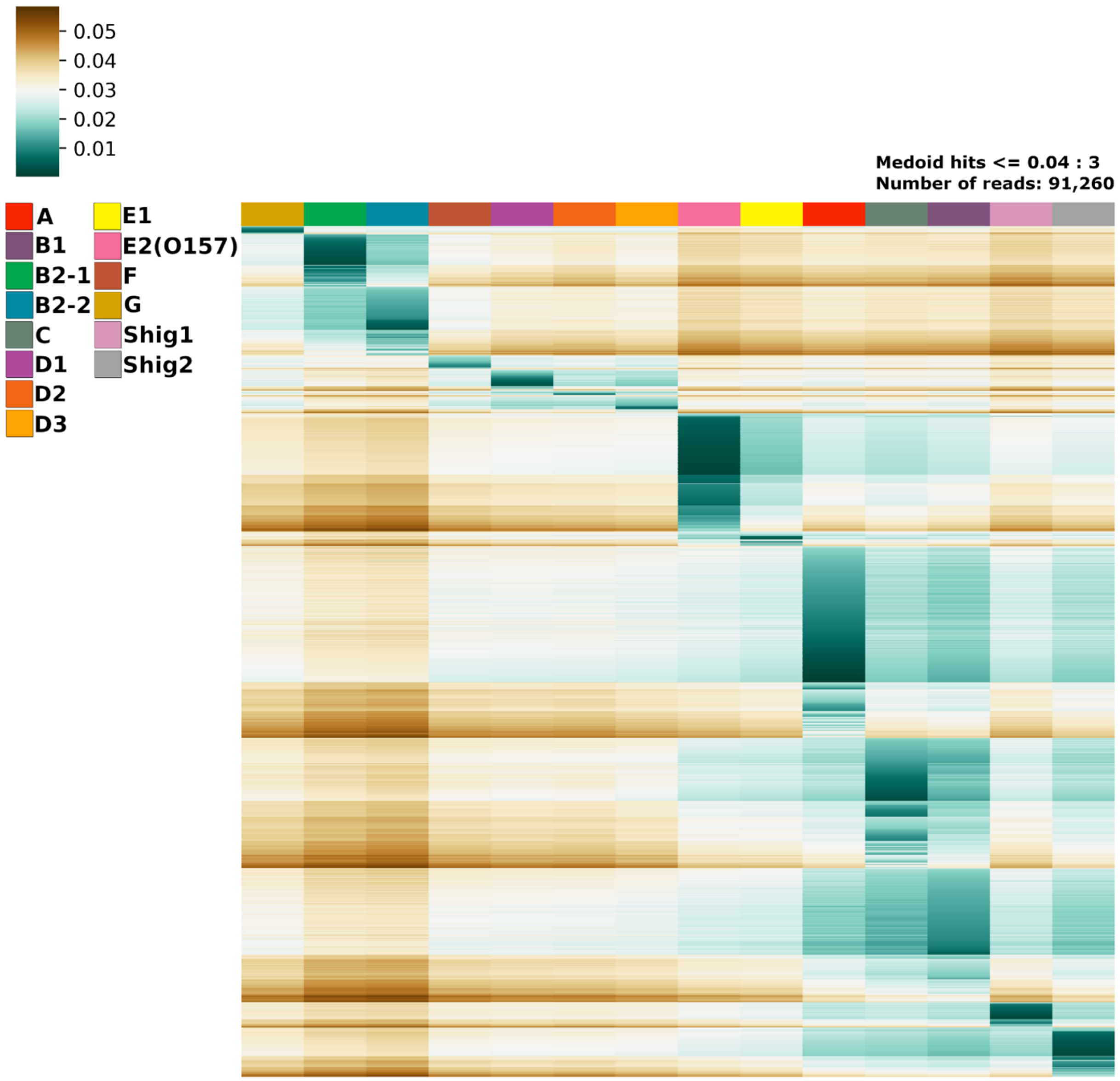

Supplementary Figure 5
Heatmap of Mash distances for 91,260 SRA reads to each of the 14 medoids. Reads were filtered to any read with a Mash distance ≤ 0.04 to at least 3 medoids.

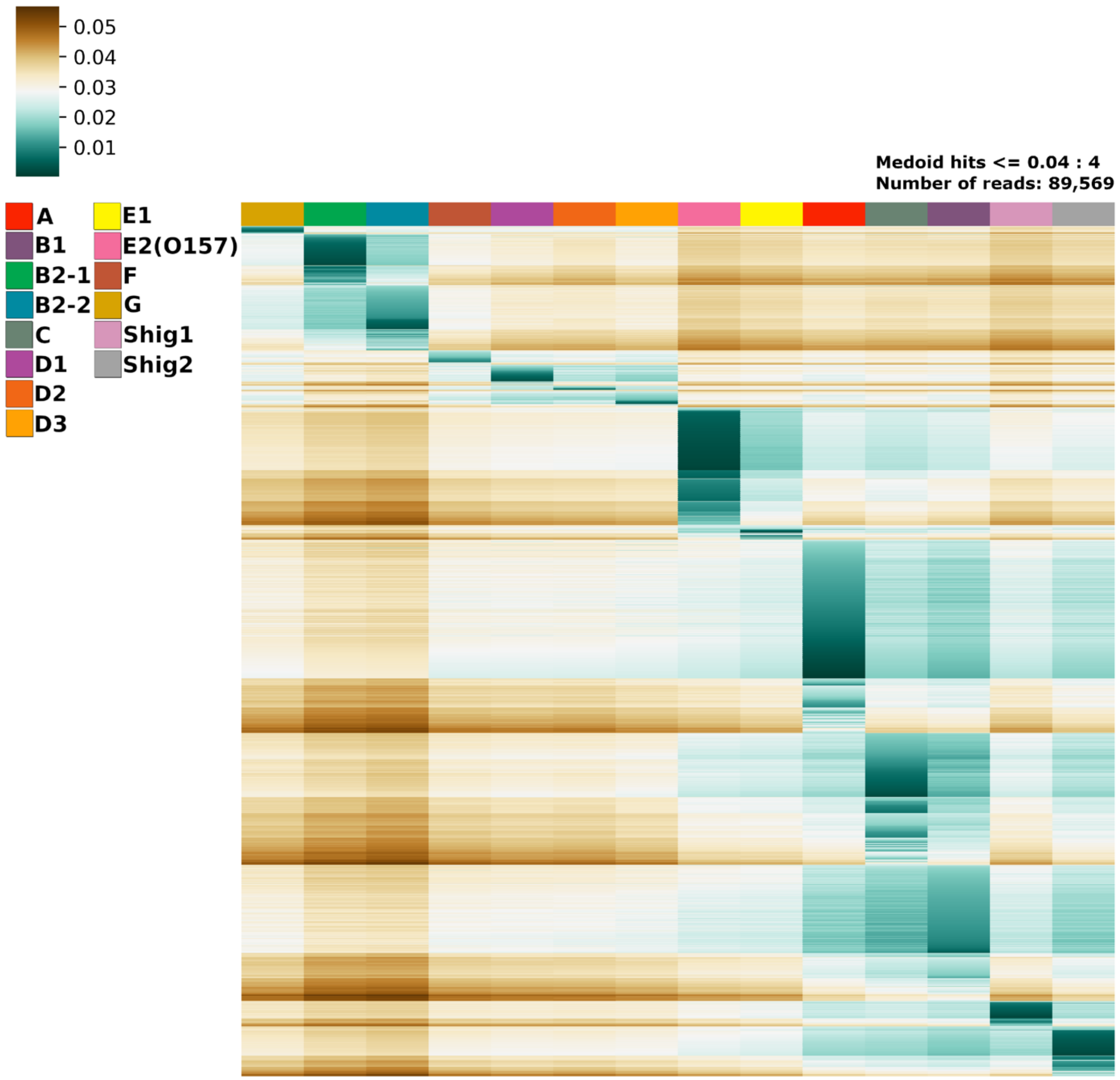

Supplementary Figure 6

Heatmap of Mash distances for 89,569 SRA reads to each of the 14 medoids. Reads were filtered to any read with a Mash distance ≤ 0.04 to at least 4 medoids.

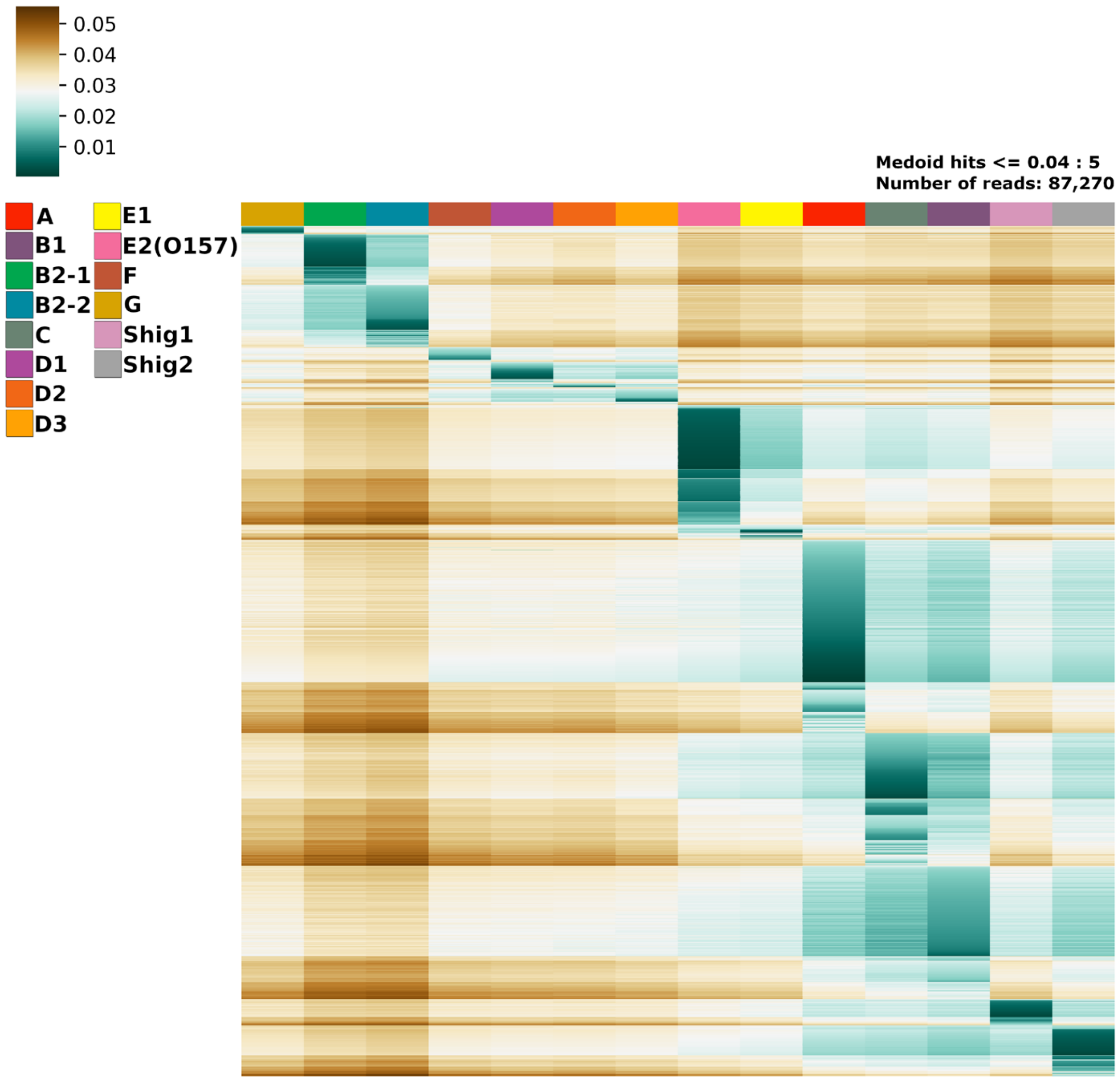

Supplementary Figure 7

Heatmap of Mash distances for 87,270 SRA reads to each of the 14 medoids. Reads were filtered to any read with a Mash distance ≤ 0.04 to at least 5 medoids.

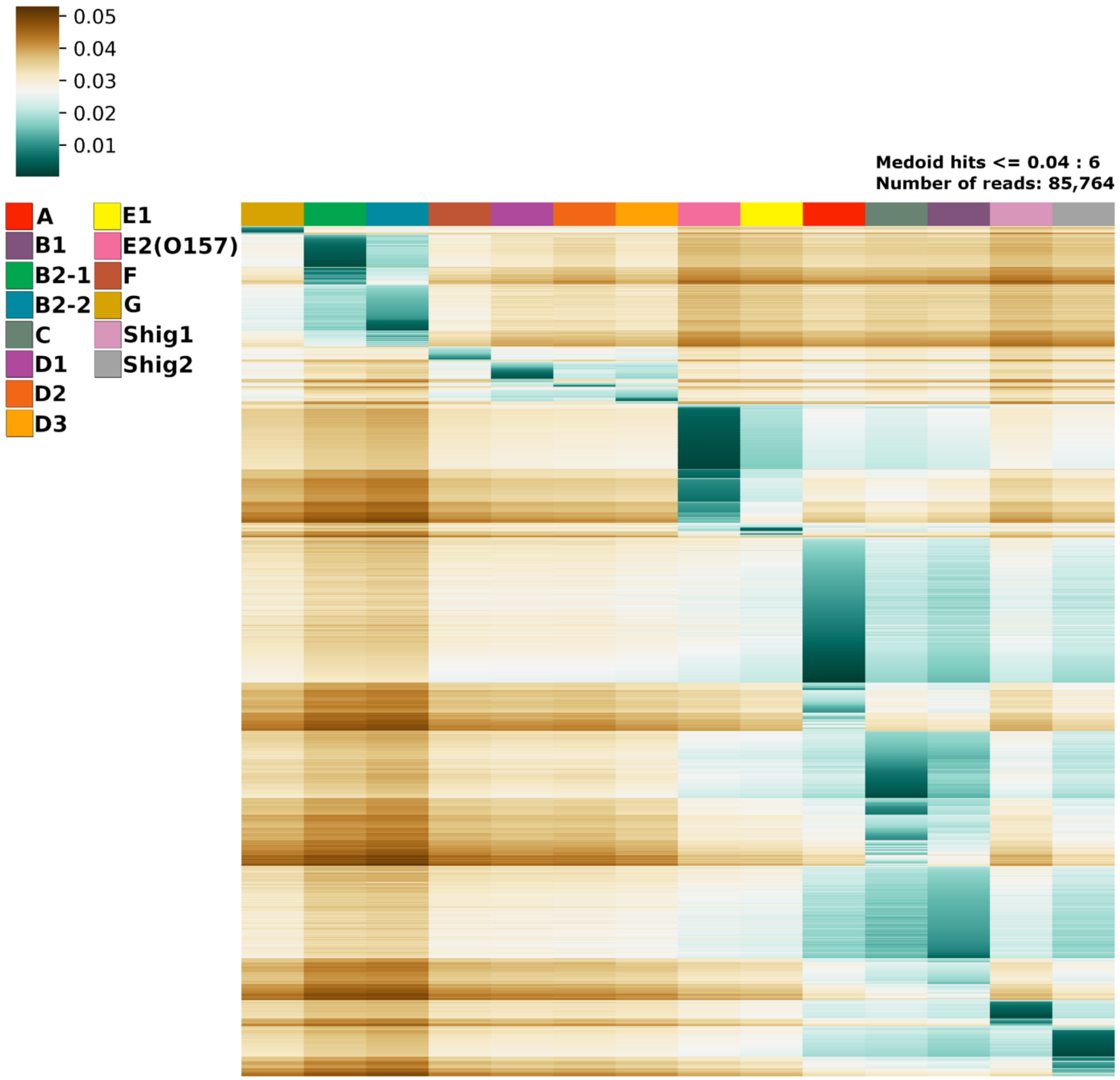

Supplementary Figure 8

Heatmap of Mash distances for 85,764 SRA reads to each of the 14 medoids. Reads were filtered to any read with a Mash distance ≤ 0.04 to at least 6 medoids.

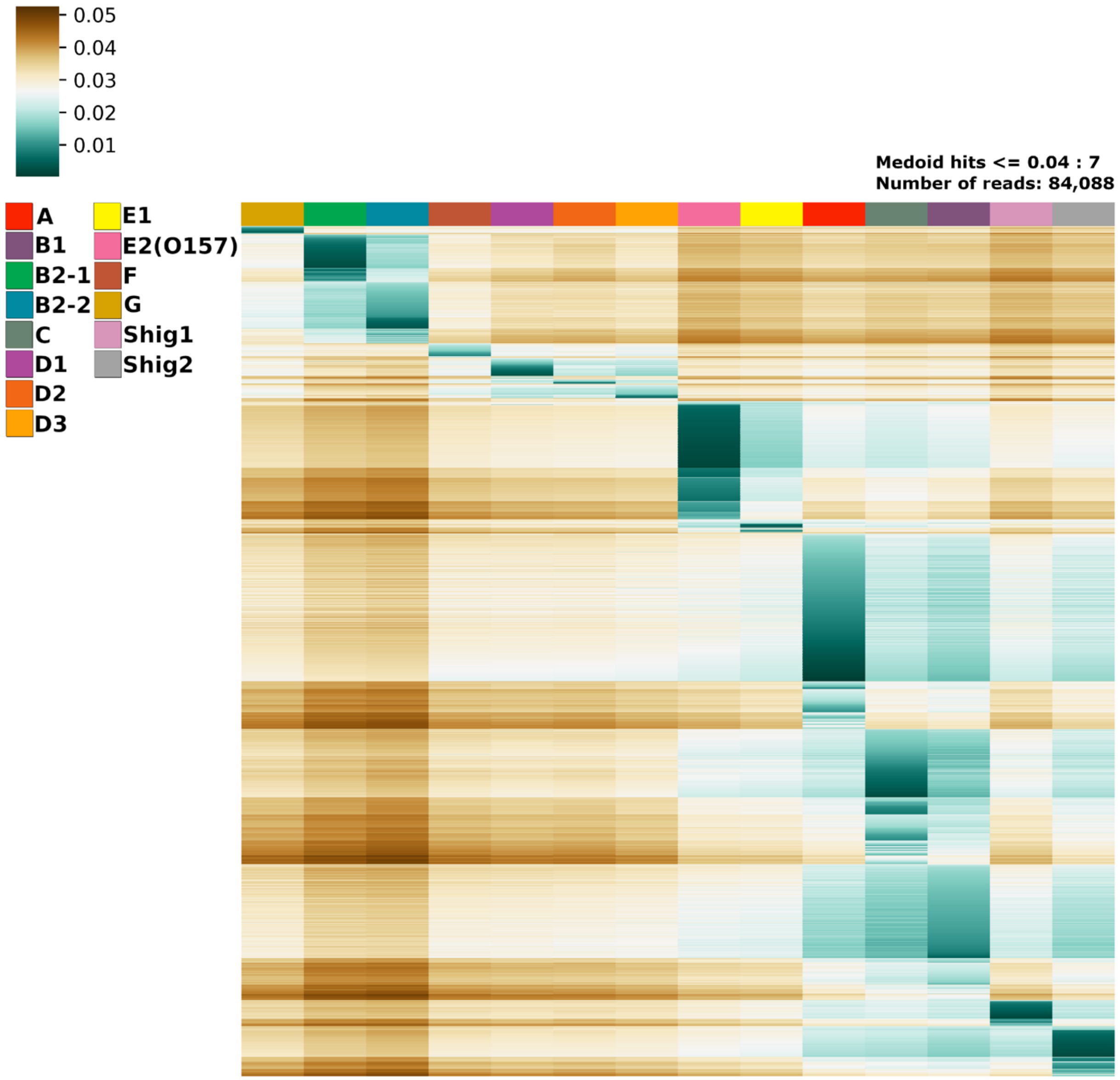

Supplementary Figure 9

Heatmap of Mash distances for 84,088 SRA reads to each of the 14 medoids. Reads were filtered to any read with a Mash distance ≤ 0.04 to at least 7 medoids.

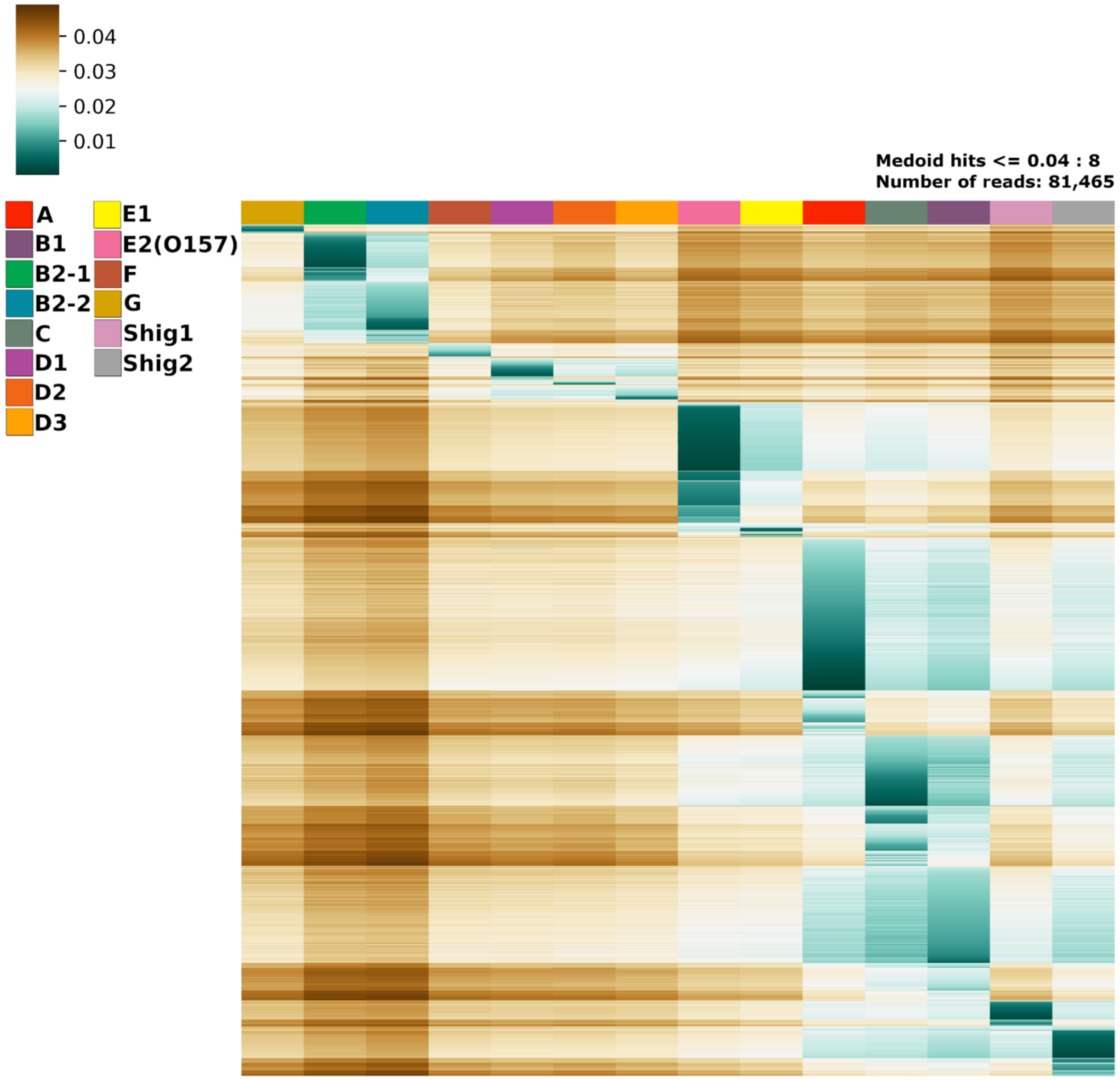

Supplementary Figure 10

Heatmap of Mash distances for 81,465 SRA reads to each of the 14 medoids. Reads were filtered to any read with a Mash distance ≤ 0.04 to at least 8 medoids.

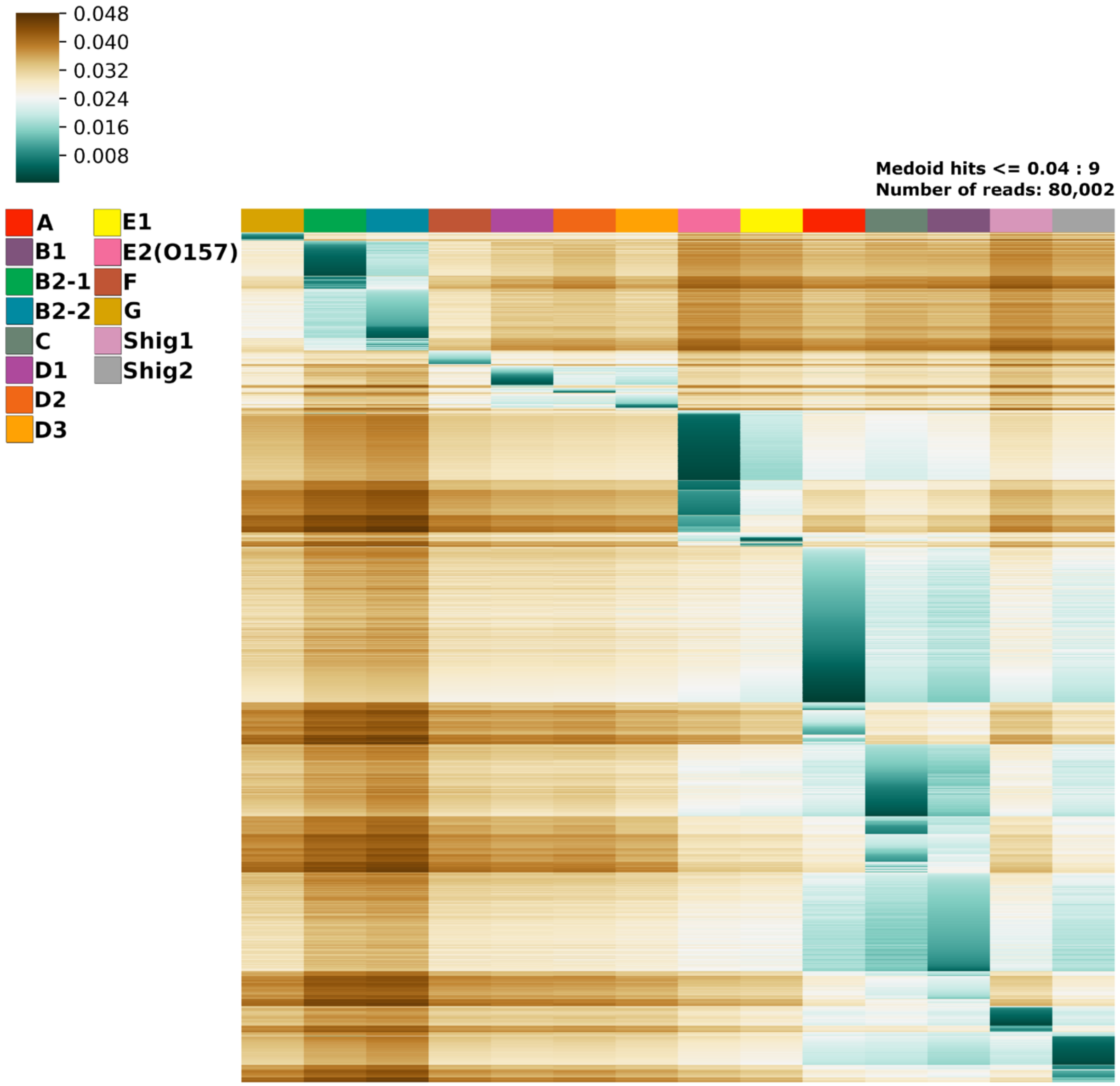

Supplementary Figure 11
Heatmap of Mash distances for 80,002 SRA reads to each of the 14 medoids. Reads were filtered to any read with a Mash distance ≤ 0.04 to at least 9 medoids.

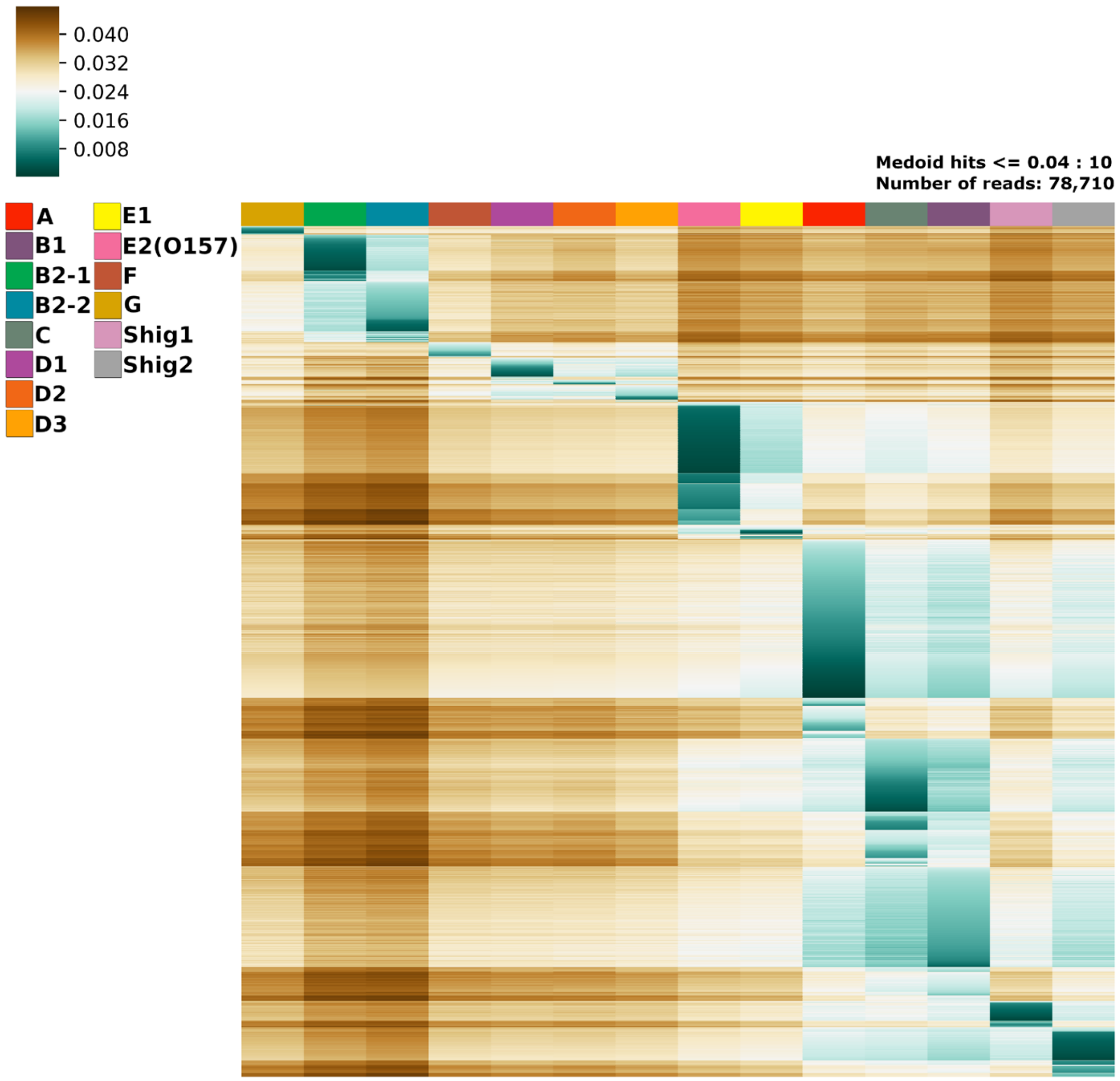

Supplementary Figure 12

Heatmap of Mash distances for 78,710 SRA reads to each of the 14 medoids. Reads were filtered to any read with a Mash distance ≤ 0.04 to at least 10 medoids.

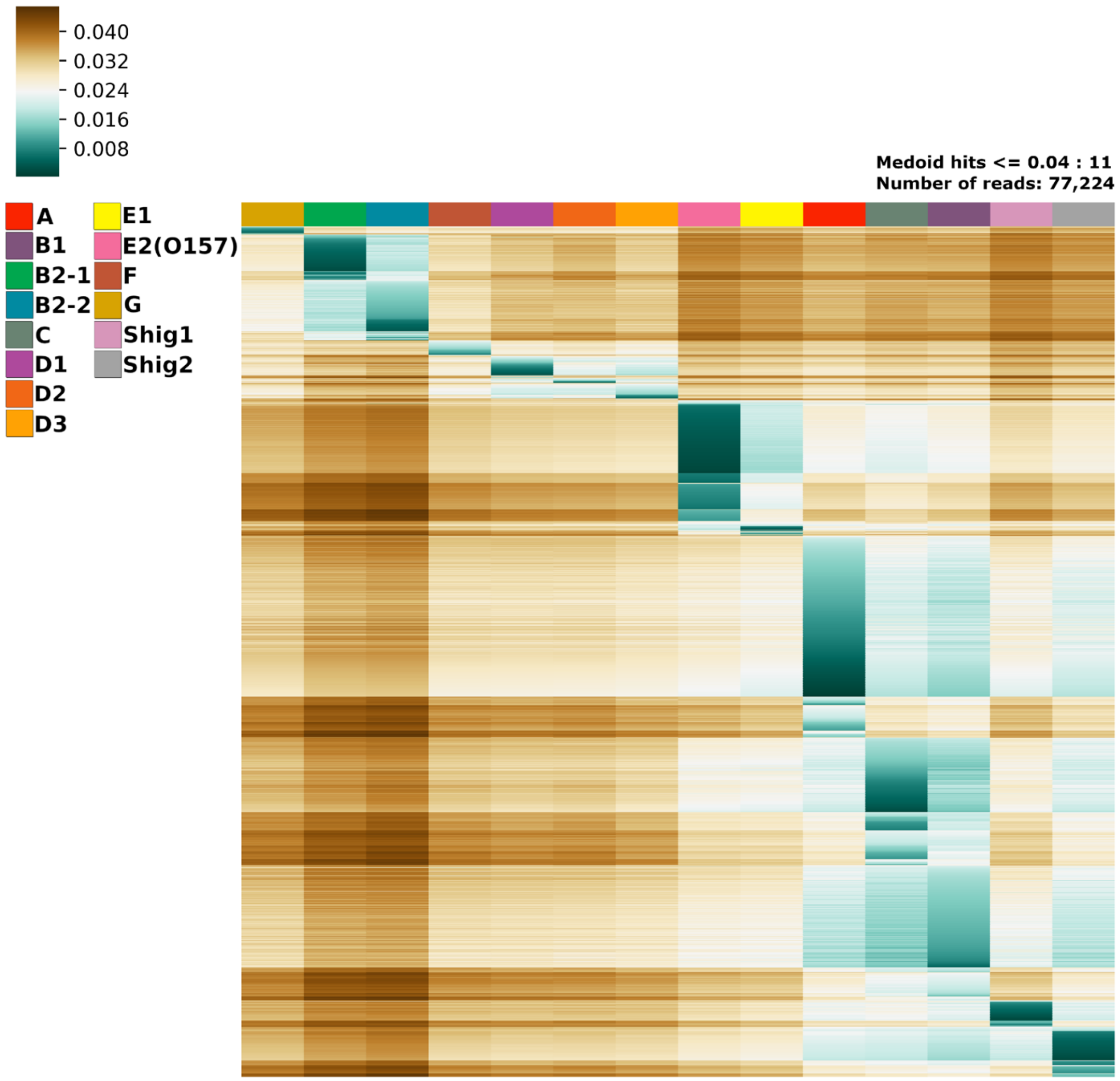

Supplementary Figure 13

Heatmap of Mash distances for 77,224 SRA reads to each of the 14 medoids. Reads were filtered to any read with a Mash distance ≤ 0.04 to at least 11 medoids.

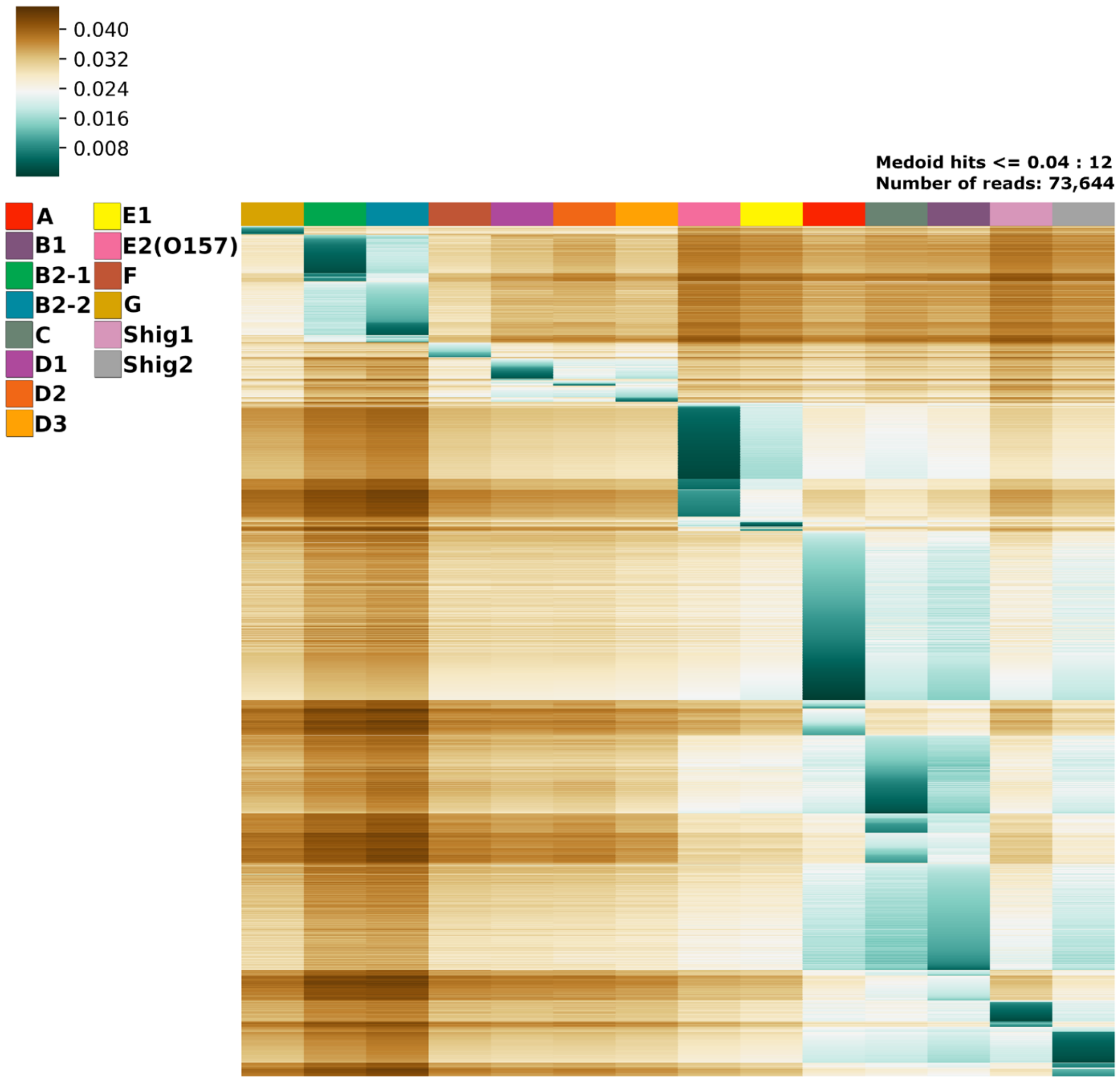

Supplementary Figure 14

Heatmap of Mash distances for 73,644 SRA reads to each of the 14 medoids. Reads were filtered to any read with a Mash distance ≤ 0.04 to at least 12 medoids.

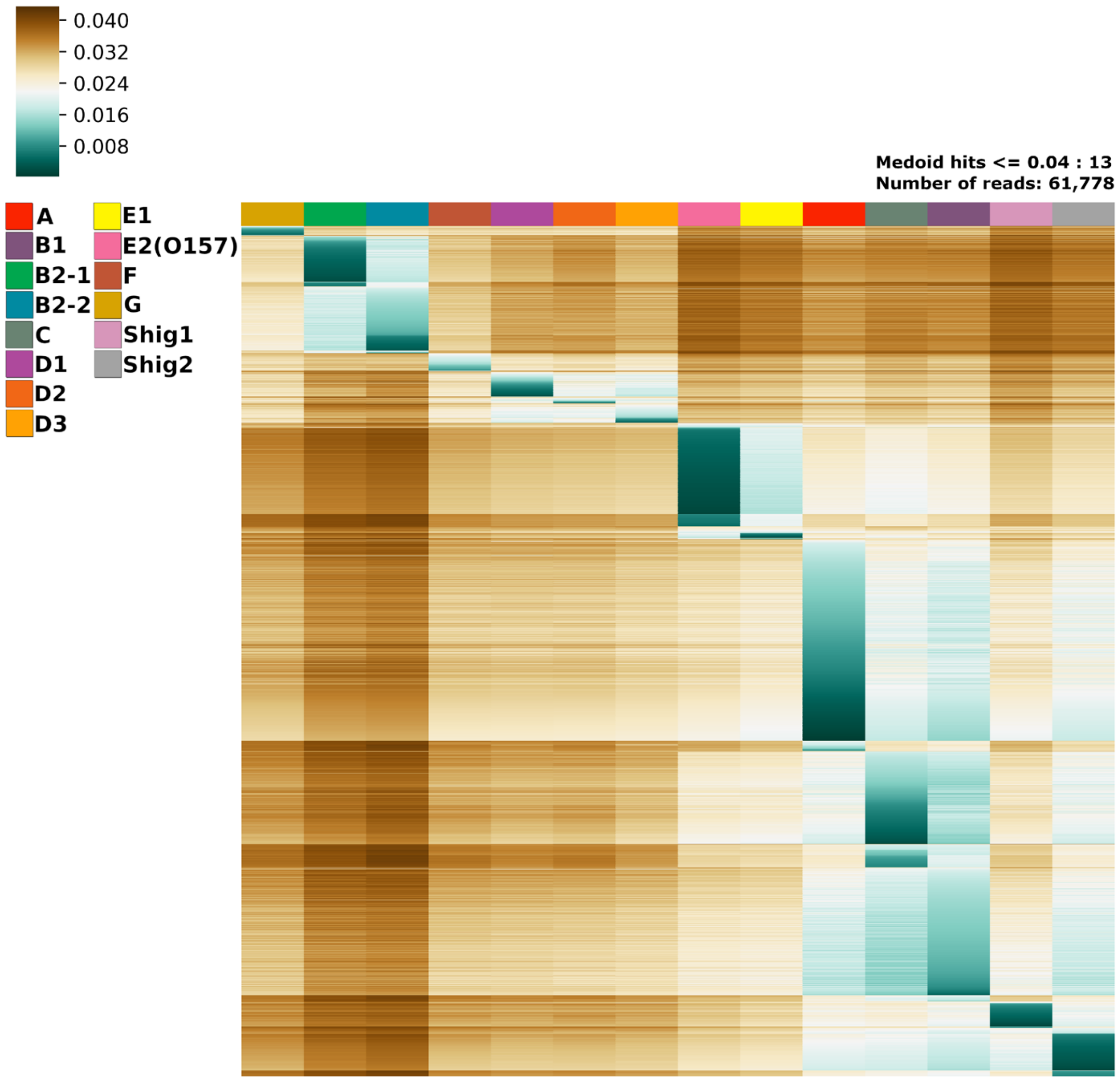

Supplementary Figure 15

Heatmap of Mash distances for 61,778 SRA reads to each of the 14 medoids. Reads were filtered to any read with a Mash distance ≤ 0.04 to at least 13 medoids.

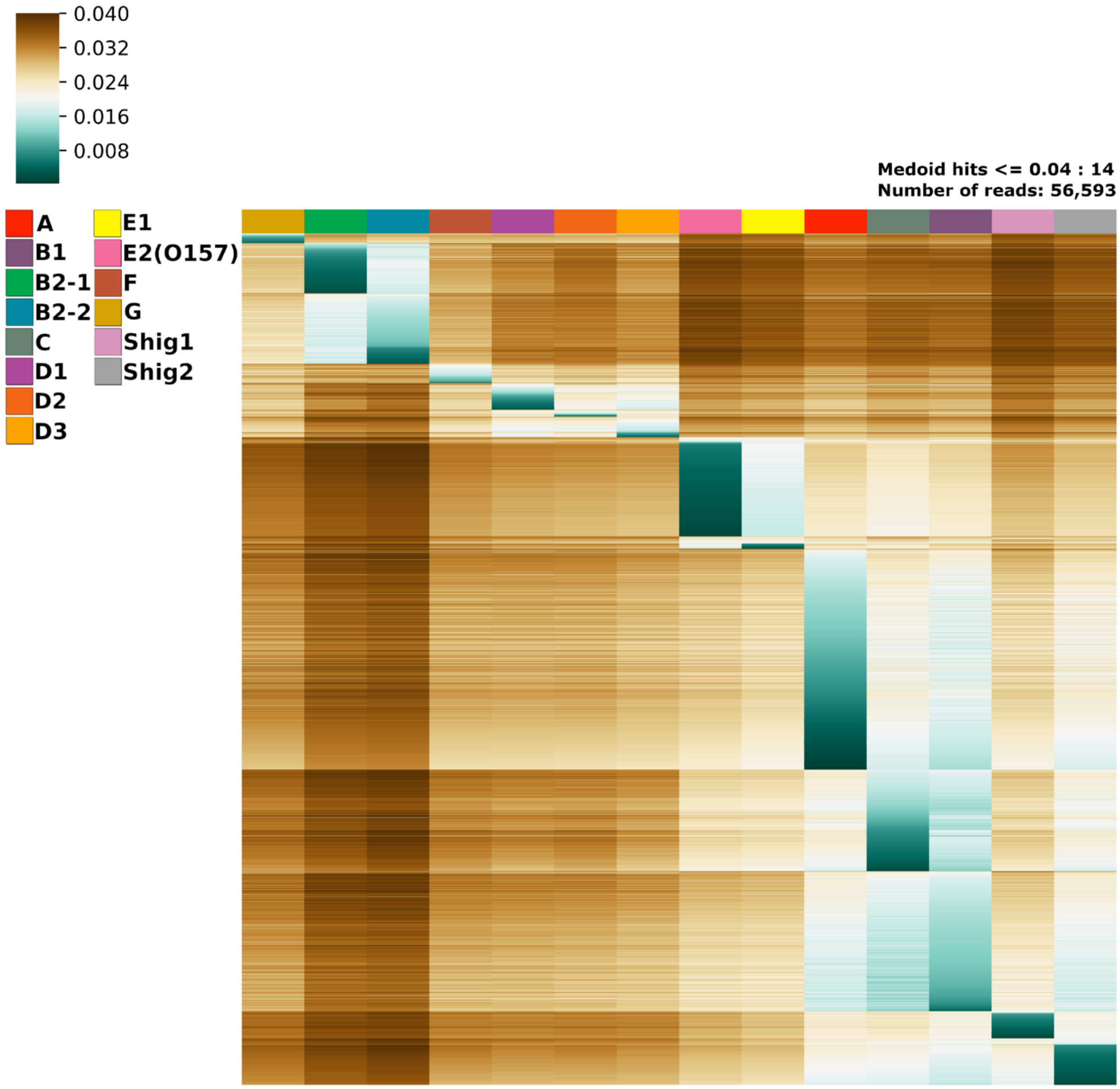

Supplementary Figure 16

Heatmap of Mash distances for 56,593 SRA reads to each of the 14 medoids. Reads were filtered to any read with a Mash distance ≤ 0.04 to all 14 medoids.

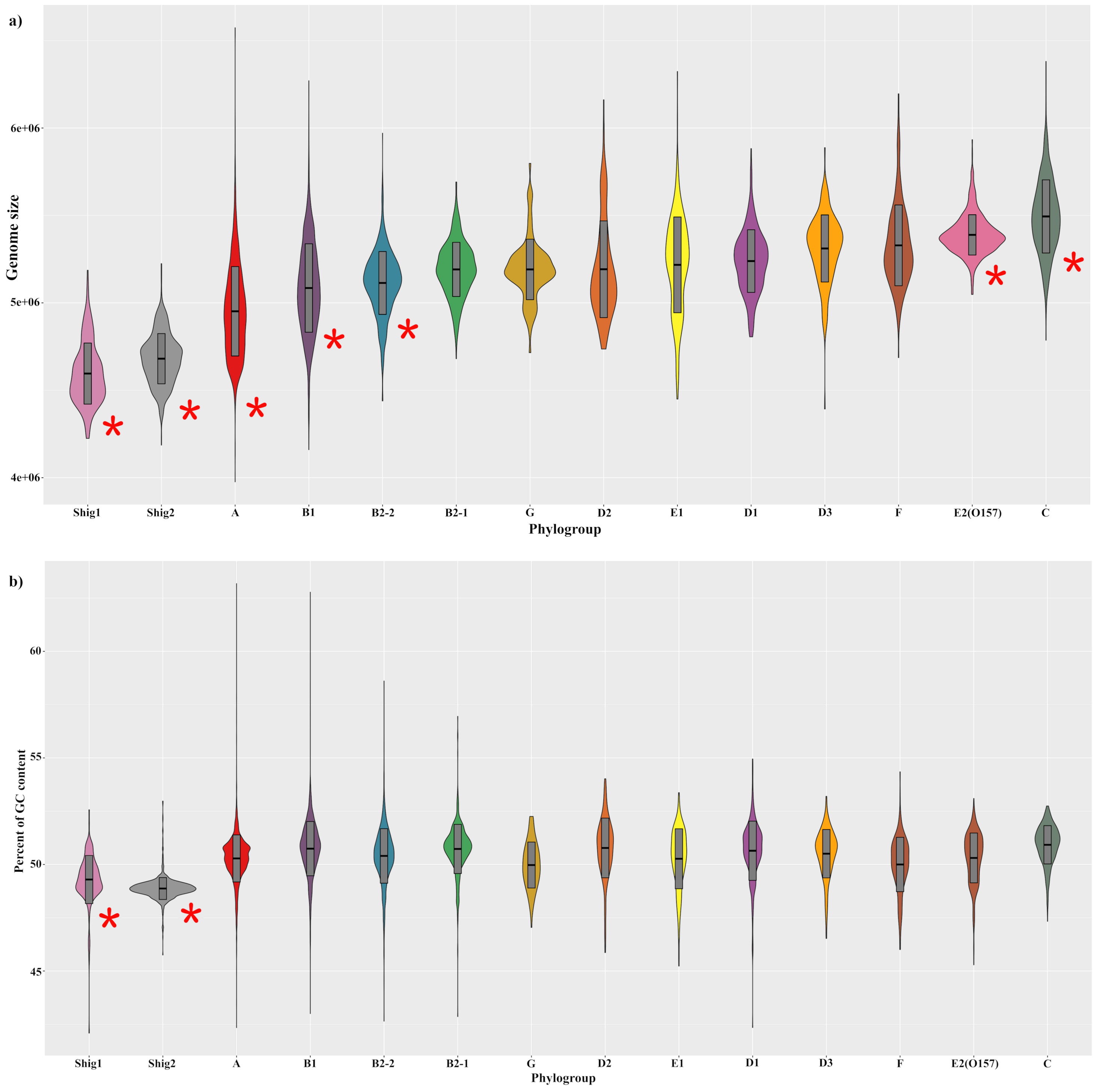

Supplementary Figure 17

Violin plots of the distribution of genome size **(A)** and genomic GC content **(B)** by phylogroup. Bar plots inside the violins represent values for mean and mean plus one standard deviation per phylogroup. Phylogroups that have values significantly different to all other phylogroups (according to F statistics test) are marked with a red asterisk.

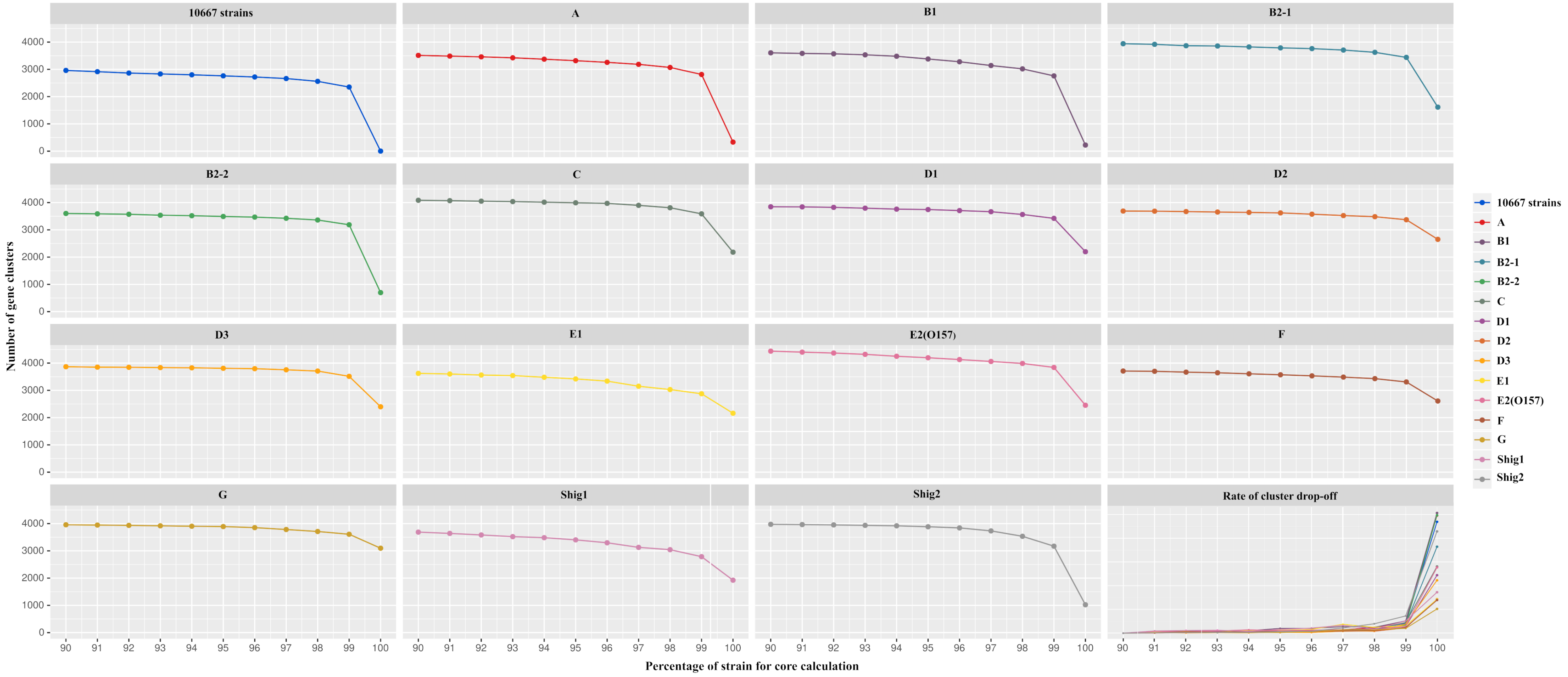

Supplementary Figure 18

Cut-offs for core genome calculation. Core genomes established at a cutoff of 90% to 100% per phylogroup. The lower right panel titled "Rate of cluster drop-off" illustrates the rate that clusters drop-off as the percent membership increases from 90% to 100% for all phylogroups and the species. The color key corresponding to each phylogroup is on the right side of the figure.

Species	Strain	Assembly Accession	Size (Mb)	Total Score	Sequence Score	Bioproject	Biosample	Phylogroup
Escherichia coli	MOD1-EC4358	GCA_002473875.1	5.34623	0.91	0.62	PRJNA230969	SAMN04913985	E2
Escherichia coli	MOD1-EC1641	GCA_002460705.1	5.20103	0.88	0.62	PRJNA230969	SAMN05607397	E1
Shigella flexneri	FC1170	GCA_002241955.1	4.59581	0.85	0.5	PRJNA393761	SAMN07340962	Shig1
Shigella sonnei	CDPH_C96	GCA_001688385.1	4.44053	0.85	0.52	PRJNA300887	SAMN04440977	Shig2
Escherichia coli	104	GCA_900096825.1	4.63813	0.99	0.95	PRJEB15352	SAMEA4444105	A
Escherichia coli	MOD1-EC3802	GCA_002516075.1	4.61361	0.92	0.86	PRJNA230969	SAMN05440280	B1
Escherichia coli	2014C-4638	GCA_003015295.1	5.77998	1	0.99	PRJNA218110	SAMN03265975	C
Escherichia coli	BIDMC 63	GCA_000692435.1	5.16234	0.99	0.97	PRJNA234151	SAMN02581284	B2-1
Escherichia coli	GSK25213	GCA_000807565.1	4.94925	0.9	0.71	PRJNA221787	SAMN03169672	B2-2
Escherichia coli	FVEC1412	GCA_000163235.1	5.23418	0.95	0.79	PRJNA39917	SAMN02595360	D1
Escherichia coli	MOD1-EC5921	GCA_002534845.1	4.7781	0.95	0.88	PRJNA230969	SAMN05452849	D2
Escherichia coli	HVH 65 (4-2262045)	GCA_000456945.1	5.31618	0.97	0.9	PRJNA186093	SAMN01885720	D3
Escherichia coli	WB61	GCA_002966755.1	4.68705	1	1	PRJNA374983	SAMN06341081	F
Escherichia coli	MOD1-EC6621	GCA_002485345.1	4.90502	0.92	0.79	PRJNA230969	SAMN04992451	G

Supplementary Table 1

A selection of metadata for the 14 medoids utilized in the analyses of this work. Additional information can be found in the metadata of the Microreact associated with this project: https://microreact.org/project/10667ecoli/c38356ec.
