# Supplementary material for: Mash-based analyses of Escherichia coli genomes reveal 14 distinct phylogroups
Source: Commun Biol. 2021 Jan 26;4:117. doi: 10.1038/s42003-020-01626-5 (PMC7838162; doi:10.1038/s42003-020-01626-5)
Supplement: Supplementary file 2 — Description of Additional Supplementary Files [file 42003_2020_1626_MOESM2_ESM.pdf]

Description of Additional Supplementary Files

File name: Supplementary Data 1

Description: Accession numbers and strain names of the 10,667 assembled genomes used in this study. In addition to their metadata, genome quality scores are also provided. This file also includes a selection of the percent cutoff and cluster information tested in this study.

File name: Supplementary Data 2

Description: SRA metadata including read name, the predicted phylogroup, and the number of phylogroup medoids a given read has a Mash distance 0.04.

File name: Supplementary Data 3

Description: Results of the ANOVA and Tukey's test for the analysis of the means for genome sizes and GC content per phylogroup.

File name: Supplementary Data 4

Description: Functional annotation using KO terms for each of the clusters identified as phylogroup unique core genes.
