# Supplementary material for: Mash-based analyses of Escherichia coli genomes reveal 14 distinct phylogroups
Source: Commun Biol. 2021 Jan 26;4:117. doi: 10.1038/s42003-020-01626-5 (PMC7838162; doi:10.1038/s42003-020-01626-5)
Supplement: Supplementary file 7 — Reporting Summary [file 42003_2020_1626_MOESM7_ESM.pdf]

nature research

Corresponding author(s): David W. Ussery

Last updated by author(s): 11/10/2020

Reporting Summary

Nature Research wishes to improve the reproducibility of the work that we publish. This form provides structure for consistency and transparency in reporting. For further information on Nature Research policies, see Authors & Referees and the Editorial Policy Checklist .

Statistics

For all statistical analyses, confirm that the following items are present in the figure legend, table legend, main text, or Methods section.

n/a	Confirmed	
☐	☒	The exact sample size (n) for each experimental group/condition, given as a discrete number and unit of measurement
☐	☒	A statement on whether measurements were taken from distinct samples or whether the same sample was measured repeatedly
☐	☒	The statistical test(s) used AND whether they are one- or two-sided<br>*Only common tests should be described solely by name; describe more complex techniques in the Methods section.*
☒	☐	A description of all covariates tested
☐	☒	A description of any assumptions or corrections, such as tests of normality and adjustment for multiple comparisons
☐	☒	A full description of the statistical parameters including central tendency (e.g. means) or other basic estimates (e.g. regression coefficient) AND variation (e.g. standard deviation) or associated estimates of uncertainty (e.g. confidence intervals)
☐	☒	For null hypothesis testing, the test statistic (e.g. F, t, r) with confidence intervals, effect sizes, degrees of freedom and P value noted<br>*Give P values as exact values whenever suitable.*
☒	☐	For Bayesian analysis, information on the choice of priors and Markov chain Monte Carlo settings
☒	☐	For hierarchical and complex designs, identification of the appropriate level for tests and full reporting of outcomes
☒	☐	Estimates of effect sizes (e.g. Cohen's d, Pearson's r), indicating how they were calculated

Our web collection on statistics for biologists contains articles on many of the points above.

Software and code

Policy information about availability of computer code

Data collection	Genomes were collected from NCBI's GenBank prokaryote summary and downloaded via Batch Entrez. Data from the SRA was downloaded via Aspera
Data analysis	Mash v2.1<br>R v3.5.1<br>Python v3.6.8<br>Cytoscape v3.7.1<br>EMBOSS v6.6.0.0<br>USEARCH/UCLUST v10.0.240<br>MAFFT v7.110<br>IQ-TREE v1.6.10<br>Count v10.04

For manuscripts utilizing custom algorithms or software that are central to the research but not yet described in published literature, software must be made available to editors/reviewers. We strongly encourage code deposition in a community repository (e.g. GitHub). See the Nature Research guidelines for submitting code & software for further information.

Data

Policy information about availability of data

All manuscripts must include a data availability statement. This statement should provide the following information, where applicable:

- Accession codes, unique identifiers, or web links for publicly available datasets
- A list of figures that have associated raw data
- A description of any restrictions on data availability

The data supporting the findings of the study are available in this article, its Supplementary Information files, or from the corresponding author upon request. Code is available on Zenodo: http://doi.org/10.5281/zenodo.4091750. A collection of metadata and our phylogenetic tree is available via: https://microreact.org/project/10667ecoli/b4431cf8. All data underlying the figures for this manuscript are also available on Zenodo with the supporting code. An animated movie of Cytoscape graphs from this analysis is available on figshare: http://dx.doi.org/10.6084/m9.figshare.13105235. Stills from this animated movie are available on figshare: http://dx.doi.org/10.6084/m9.figshare.11473308.

Field-specific reporting

Please select the one below that is the best fit for your research. If you are not sure, read the appropriate sections before making your selection.

[x] Life sciences   [ ] Behavioural & social sciences   [ ] Ecological, evolutionary & environmental sciences

For a reference copy of the document with all sections, see nature.com/documents/nr-reporting-summary-flat.pdf

Life sciences study design

All studies must disclose on these points even when the disclosure is negative.

Sample size	No sample size calculation was performed. This research was based on all available Escherichia and Shigella genomes in GenBank on June 26, 2018. The purpose of this study was to present an up to date and in-depth analysis of the Escherichia coli species using all genome sequences labeled Escherichia or Shigella that were of sufficient quality.
Data exclusions	The dataset was filtered first by genome quality score which removed genomes of very low quality. A genome size restriction was also applied to avoid genomes that had been genetically manipulated so much that the resultant genome size was outside the naturally occurring range. After these two filters, genomes were removed that did not fall within a 98.5% distribution of Mash distances for both chosen type strains of Escherichia coli and Shigella. All genomic .fna files were ran through MD5 checksum to identify identical genomes and retain a single copy.
Replication	This dataset was analyzed using multiple cutoffs and statistical distributions of Mash distances for all strains to the type strains utilized in this study. Once the cutoffs were chosen, pangenomic analysis was performed as described in the manuscript. The majority of the genomes utilized in this study had a consistent classification.
Randomization	Randomization was not relevant to this study as all genomes were subject to the same genomic analyses in order to fully characterize the dataset.
Blinding	Once the genomes of the analysis were selected, their associated metadata (including accession numbers) was completely ignored and all genomes were identified by MD5 checksum. Phylogroup association data was not collected until after the analysis was complete to identify our predicted phylogroups.

Reporting for specific materials, systems and methods

We require information from authors about some types of materials, experimental systems and methods used in many studies. Here, indicate whether each material, system or method listed is relevant to your study. If you are not sure if a list item applies to your research, read the appropriate section before selecting a response.

Materials & experimental systems

n/a	Involved in the study	
[x]	[ ]	Antibodies
[x]	[ ]	Eukaryotic cell lines
[x]	[ ]	Palaeontology
[x]	[ ]	Animals and other organisms
[x]	[ ]	Human research participants
[x]	[ ]	Clinical data

Methods

n/a	Involved in the study	
[x]	[ ]	ChIP-seq
[x]	[ ]	Flow cytometry
[x]	[ ]	MRI-based neuroimaging
